# Sustainability-based comparative stability of oxaliplatin plus leucovorin and 5-fluorouracil in infusion bags with application to plasma and colonic media samples

**DOI:** 10.1038/s41598-025-02079-8

**Published:** 2025-05-23

**Authors:** Hadir M. Maher, Salma Mahmoud Mohamed, Ekram M. Hassan, Amira Fawzy El-Yazbi

**Affiliations:** Pharmaceutical Analytical Chemistry Department, Faculty of Pharmacy, University of Alexandria, Elmessalah, 21521 Alexandria Egypt

**Keywords:** HPLC-DAD, FOLFOX, Stability, Infusion bags, Normal saline, 5% dextrose, Greenness, Blueness, Whiteness, Cancer, Chemistry, Materials science

## Abstract

FOLFOX is widely used in the treatment of colon cancer. The combination of oxaliplatin (OXA), 5-fluorouracil (5-FU), and leucovorin (LV) is administrated according to strict protocols. Based on incompatibility studies, LV and OXA are supplied concurrently via intravenous infusion, followed by intravenous administration of 5-FU. This study aims at determining the stability of the binary mixture of OXA and LV diluted in infusion bags containing either 5% dextrose or normal saline compared with their single solutions besides single solutions of 5-FU at two temperatures (room temperature and refrigeration) for two weeks. HPLC diode array detection method was developed and validated to simultaneously determine the three drugs. Stability studies in 5% dextrose solution revealed that mixtures of OXA and LV kept at room temperature showed chemical degradation for OXA, with better stability for refrigerated solutions. Generally, single solutions showed considerable stability. Using normal saline, a combination of OXA/LV showed relative stability of LV over the 15 days of study with marked degradation of OXA. 5-FU was relatively stable in 5% dextrose and normal saline at both temperatures. Method’s applicability was extended to the determination of the cited drugs in plasma and colonic media samples. Method’s sustainability, blueness, and whiteness were investigated.

## Introduction

Colon cancer (CC) is the third most frequent malignancy and is the second major cause of cancer mortality globally. Lymph node invasion is a prominent prognostic factor in localized CC, with 5-year overall survival (OS) rates of 99% and 70% for patients with stage I and II CC, respectively, versus just 45 to 65% for stage III patients, who account for around 35% of CC at diagnosis^1^. Management protocols for CC usually rely on treatments including surgery, chemotherapy, radiation therapy, and targeted therapy. Selection of a particular plan is based on a number of variables, such as the cancer’s stage, potential side effects, the patient’s preferences, and general health^2^.

Since the late 1980s, adjuvant chemotherapies have become more significant for various stages of cancer and are administered in accordance with conventional clinical criteria^3^. The treatment of CC has involved the use of five different regimens: FOLFIRI (5-fluorouracil (5-FU), leucovorin(LV), and irinotecan); FOLFOXIRI (LV, 5-FU, oxaliplatin (OXA), and irinotecan); Cape-Ox (capecitabine and OXA); FOLFOX (5-FU, LV, and OXA); besides combination therapy of 5-FU and LV. Yet the serious side effects including severe diarrhea with the use of FOLFIRI, FOLFOXIRI^4^, and hand-foot syndrome with severe nausea/vomiting generated from capecitabine in Cape-Ox contributed to their current limited use^5^. Since the early 1990s, the protocols which have been continued to be commonly employed for the treatment of CC are FOLFOX and 5-FU/ LV binary combinations^6^.

In 2004,the MOSAIC ( Multi Omic Spatial Atlas in Cancer) research established the FOLFOX regimen as a standard treatment for stage III CC^4,7^. The inclusion of OXA to the doublet combination of 5-FU/LV leads to a considerable improvement in response, with an extension of progression-free survival (PFS) and overall survival (OS)^8,9^. Nowadays, FOLFOX is commonly used as adjuvant therapy for patients with high risk of recurrence (stages II and III) of primary CC or metastatic colorectal cancer. However, it is noteworthy to mention that despite FOLFOX is considered as the relatively safest protocol, OXA-induced neuropathy is the most significant negative impact associated with this protocol particularly for old patients^10^. This therapy is administered intravenously every two weeks based on patients’ response and tolerance.

5-FU, a uracil analog, is a chemotherapeutic agent used for the management of CC as well as different types of cancers. Being an antimetabolite, it blocks cancerous cells’ proliferation by interfering with different stages of DNA and RNA synthesis^11^. Thus, it serves as the most widely utilized medication for the treatment of stages II and III metastatic CC.

Calcium leucovorin (LV), also known as calcium salt of folinic acid, is commercially available as a 1:1 racemic combination of the l- and d- isomers with the levo-isomer being the pharmacologically active form. It is used in anticancer FOLFOX protocol to increase the cytotoxic effects of 5-FU through stabilizing its binding to the thymidylate synthase enzyme^12^. Accordingly, treatment of CC with adjuvant 5-FU/LV combination is more effective than 5-FU monotherapy in terms of overall survival and response rate^9^.

**Fig. 1:**
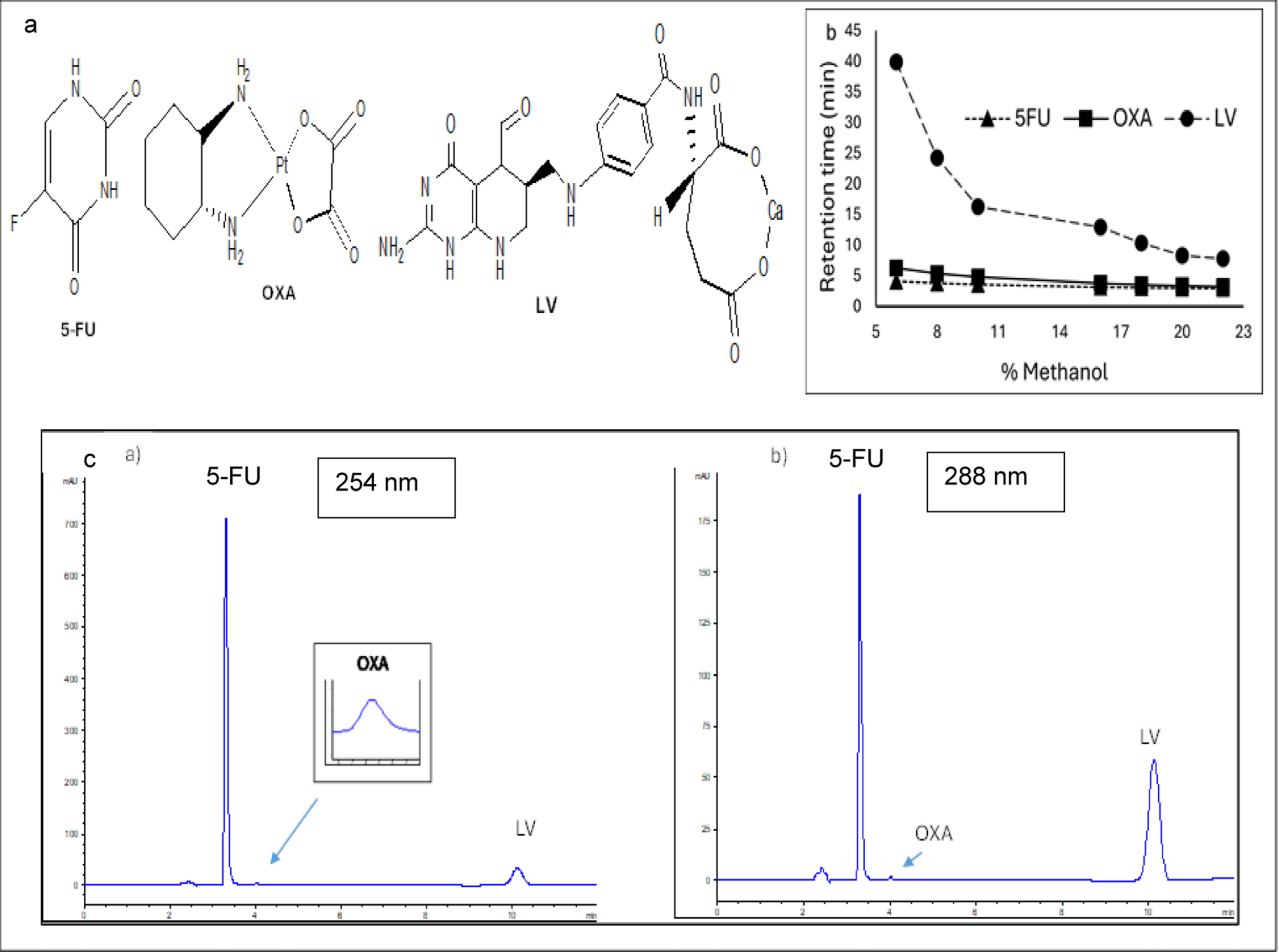
(**a**) Chemical structures of the studied compounds, 5-Fluorouracil (5-FU), Oxaliplatin (OXA), and Leucovorin (LV). (**b**) Effect of %methanol in the mobile phase on the retention time 5-FU, OXA, and LV. (**c**) Typical chromatograms of a studied mixture of 5-FU (50 µg/mL), OXA (3 µg/mL), and LV (14.4 µg/mL) using optimized chromatographic conditions measured at 254nm, and 288 nm.

Oxaliplatin (OXA) is a third-generation anticancer platinum molecule. Pt based medications are reported to have diverse mechanisms of action. Cisplatin and its platinum analogs, carboplatin and OXA, are widely used chemotherapeutic agents of different clinical implementation. Cisplatin and carboplatin are used primarily in the management of breast and lung cancer, while OXA is mainly used in the treatment of colorectal and gastrointestinal malignancies. It was shown that OXA exhibits its anticancer effect by inducing ribosome biogenesis stress rather than DNA damage caused by the other two platinum drugs, cisplatin and carboplatin^13^. These medications often create cross-linking adducts in plasma during non-enzymatic transformation, resulting in the prevention of DNA transcription and replication^14^. It was also previously revealed that angiogenin could be a possible target of the platinum antineoplastic drug OXA^15^. Although the precise mechanism of synergism of 5-FU and OXA is very complex, experimental data indicates that OXA may be able to block or downregulate dihydropyrimidine dehydrogenase, which would slow down 5-FU’s catabolism^16^. Chemical structures of 5-FU, OXA, and LV are shown in Fig. 1a.

FOLFOX is currently not available in an all-in-one dosing form. Drugs (LV and OXA) are available as a sterile lyophilized powder that must be reconstituted and diluted before use^17^. In practice, each medicine is supplied individually following a tight chronological order. LV and OXA are often supplied concurrently over a two-hour period via intravenous (IV) infusion using a Y-shaped tube positioned directly before the injection site to avoid a long-term combination of both drugs since LV solution contains sodium chloride and sodium hydroxide for pH correction to 6.5–8.5. However, the alkaline pH of the LV solution is detrimental to the stability of OXA, which undergoes alkaline hydrolysis^18^. In addition, chloride salts may have a negative impact on OXA’s stability due to its quick conversion to reactive species (monochloro-, dichloro-, and diaquoplatinum)^19^. Although, it is suggested to dilute OXA with 5% dextrose (D5W) instead of normal saline (NS), a risk of phlebitis and venous discomfort may arise^20,21^. Moreover, D5W itself may be contraindicated for diabetic patients administering OXA. On the contrary, other studies suggested the compatibility of solutions of OXA and LV mixed together up to 4 h with no signs of instability^22^. As a matter of fact, solution mixtures of both drugs are likely to occur in the combined tubing of the administration set. Following the 2 h administration of OXA and LV ,5-FU is administrative intravenously as a bolus, followed by continuous infusion. This separated administration is based on the incompatibility of the highly alkaline 5-FU solution with OXA. Moreover, it was reported that high concentrations of LV induce catheter obstruction following 5-FU perception^23,24^.Despite all previous precautions, a great chance of the concomitant mixing of OXA and LV in the junction of Y- shaped tube is still unavoidable. Based on the significant effect of pH on solutions’ stability, it is noteworthy to mention that solutions of binary mixtures of OXA/LV recorded a pH of 5.5 $\:\pm\:$ 0.5, while those of 5-FU solutions exhibited pH values of 9.15 $\:\pm\:$ 0.2.

Since therapeutic protocols rely on diluting medications using NS or D5W as universal diluents and since the drug diluents could play an important role on the stability of drugs, it was thought of extending the stability studies to mixtures’ solutions diluted in NS/ D5W in comparison with their corresponding single diluted solutions. Literature review revealed that some stability studies of the single cited medications in NS/D5W have been reported. The stability of 5-FU in NS^25–29^and in D5W^27,29^has been studied. Also, OXA stability in D5W^20,30–32^ and in NS, Ringer lactate, Dianeal^®^ PD4, and phosphate buffer^20^ has been published. LV studies in NS^33–35^ and D5W^33,35–37^were also reported.

Considering the aforementioned incompatibility between 5-FU and LV^23,24^, in addition to the sequential order in administering the drugs in clinical protocol^38^, our study will focus on the stability of a binary mixture of OXA / LV as a possible combination that could be found in actual practice. To our knowledge, no previous reports have been found in the literature so far dealing with the stability of OXA/LV in NS/D5W. Accordingly, the study aims at measuring the stability of binary admixtures of OXA/LV in comparison with their single solutions along with single 5-FU solutions.

In this study, HPLC supported with DAD detection method was developed and validated for the simultaneous determination of the three drugs. The applicability of the method was extended to stability studies of the cited compounds dispensed in infusion bags diluted with either D5W or NS at two different temperatures 25 ^o^C and refrigeration (2–8 °C). Moreover, analysis of the plasma samples as well as colonic media samples spiked with the studied medications was also performed. Only one report was found in literature dealing with simultaneous determination of the three drugs using HPLC-DAD^17^. However, the long runtime (> 68 min ) of this method limits its throughput. Thus, our study also comprises evaluation of the degree of greenness, blueness and whiteness of the proposed method compared with the published one^17^.

## Materials and methods

### Instrumentation

Chromatographic analysis was performed using HPLC Agilent 1200 Infinity series including a vacuum degasser (G 7122 A), automatic sampler, a quadruplicate pump (G 1328B), and photodiode array detector (DAD) (G1315 C/D). The software used for the measurement was Agilent ChemStation Technology, (Santa Clara, CA, USA). Separations were carried out using Agilent Zorbax Eclipse plus C18 analytical column with the following dimensions: 4.6 × 250 mm, 5 μm particle size. pH meter JENWAY (Model 3505) was used to adjust the pH of the solutions. Nitrogen evaporator (vacuum evaporator (Christ^®^),Germany) was used for preparation of biological samples.

### Material and reagents

HPLC grade methanol ( Fisher chemicals) and trifluoroacetic acid 99% (ADVENT CHEMBIO PVT.LTO, Lot# F22K051, India) were used in the study. Deionized water was filtered through a 0.45 μm membrane filter (Sartorius AG 37070 goettingen, Germany) before being used. 5-Fluorouracil for injection 500 mg/10mL, Utoral^®^, (Lot#220145, Hikma specialized pharmaceuticals, Cairo, Egypt), oxaliplatin for injection 100 mg/20mL, Oxaliplatin ^®^, (Lot# 220038, Hikma specialized pharmaceuticals, Cairo, Egypt), leucovorin for injection 100 mg/10mL, Calcifolinon^®^, (Lot# 23117001, Global Pharmaceutical Industries(2), 6th October City, Egypt) were purchased from the local market. Trimediazine (internal standard, Medizen Pharmaceutical Industries, Borg El Arab industrial 4th Zone Block 2, Alexandria, Egypt) was used in the study. Dextrose 5%(Lot#232484 ,MUP, Egypt), 0.9% sodium chloride (Lot# 3G53P, Egypt Otsuka pharmaceutical Co, 10th of Ramadan City, Egypt) were involved in the stability study.

### Chromatographic conditions

Separations were performed using a gradient elution mode with wash and equilibration stages. The mobile phase was made up of deionized water which pH was adjusted with diluted trifluoroacetic acid (TFA) (0.0001%, v/v) (solvent A) and methanol (solvent B), the mobile phase was filtered with 0.45 μm membrane filter (sartorius AG 37070 goettingen, Germany) before being used. Final analysis was carried out with an injection volume of 20µL and 1 mL/min flow rate at room temperature (25 °C). Detection was carried out with DAD using multiple wavelengths of 266, 254, and 288 nm for the detection of 5-FU, OXA, and LV, respectively. Peak purity plots were obtained by DAD.

### Preparation of solutions and construction of calibration curve

Solutions of 5-FU, OXA, and LV (1 mg/mL) were prepared using a diluting solvent of HPLC-grade water and methanol (90/10, v/v). The stock solutions were diluted with the same solvent system to prepare standard solutions of concentrations 0.125–50 µg/mL, 0.3–125 µg/mL, and 0.2–100 ug/mL of 5-FU, OXA, and LV, respectively. Solutions were freshly prepared and filtered by passing through 0.45 μm syringe filter (Alllabware Group, China) prior to being injected into the HPLC system. A stock solution of trimediazine, being used as the internal standard (IS) in the analysis of biological samples, was prepared at a concentration of 0.5 mg/mL in the same diluting solvent. Standard solutions were chromatographed under the optimized conditions (Chromatographic conditions). The obtained peak areas (stability study) or peak area ratios (analysis of biological samples) were separately related to the corresponding concentrations to obtain the calibration curve of each compound, and the regression equations were then calculated.

### Preparation of laboratory prepared mixtures

Laboratory prepared mixtures with different concentration ratios of the studied drugs were prepared within the linearity range as follows: (5 µg/mL 5-FU, 25 µg/mL OXA, and 8 µg/mL LV), (50 µg/mL 5-FU, 3 µg/mL OXA, and 14.4 µg/mL LV), ( 24 µg/mL 5-FU, 8 µg/mL OXA, and 8 µg/mL LV ), (10 µg/mL 5-FU, 10 µg/mL OXA, and 30 µg/mL LV), and (5 µg/mL 5-FU, 10 µg/mL OXA, and 5 µg/mL LV).

### Stability study of prepared infusion bags

#### Preparation of infusions

Medication concentrations involved in the prepared study were calculated for an individual weighing 70 kg with a body surface area of 1.8 m^2^. Calculations were based on the most frequently used therapeutic regimen in clinical practices^38^ as follows: OXA 85 mg/m^2^, LV 400 mg/m^2^, and 5-FU 2400 mg/m^2^ corresponding to150 mg OXA, 720 mg LV, and 4500 mg 5-FU. In this study, the stability of the binary combination OXA and LV as well as 5-FU, OXA, and LV alone were assessed in infusion bags containing either NS or D5W.

Accordingly, the experimental design was based on preparation of final concentrations of 5000 µg/mL 5-FU, 300 µg/mL OXA, and 1440 µg/mL LV in the infusion bags. This was performed by dilution of commercial vials as follows: appropriate volumes of each of 5 mg/mL OXA (0.6 mL ) and 10 mg/mL LV (1.44 mL) were taken in infusion bags in case of binary combinations. Separate volumes of 50 mg/mL 5-FU (1 mL) / 5 mg/mL OXA (0.6 mL) /10 mg/mL LV (1.44 mL) were used to study the individual stability.

Final dilutions to 10 mL were then made with either NS or D5W as a diluent.

#### Test groups

Binary mixtures of OXA (300 µg/mL) and LV (1440 µg/mL) besides single drugs of 5-FU (5000 µg/mL), OXA (300 µg/mL), or LV (1440 µg/mL) were prepared in a series of infusion bags containing either D5W (set 1) or NS (set 2). For each of the two sets, the stability was assessed at two temperatures, room temperature (25 °C) and refrigeration (2–8 °C). Each infusion bag was covered with polyethylene wrap and kept protected from light.

#### Stability assay and sampling timeline

Separate volumes of 100 µL were taken from each infusion bag at different time intervals, days 0 (onset of preparation) ,2,4,7,9,11,14,16 (D5W, set 1 ) and days 0,2,5,7,9,12,14,15 (NS, set 2).These volumes were diluted in a series of 10 mL volumetric flasks using a diluting solvent of water and methanol (90/10, v/v).Diluted solutions were then chromatographed under the optimized conditions (Chromatographic conditions) and the found concentrations for the studied drugs were calculated from the corresponding regression equations.

### Analysis of biological media

The animal study has been designed, and the results have been reported in accordance with the ARRIVE Guidelines. All animal experiments were conducted according to an experimental protocol approved by the Institutional Animal Care and Use Committee at Alexandria University (Serial Number 0620234121151) and in compliance with the Guide for the Care and Use of Laboratory Animals of the Institute for Laboratory Animal Research of the National Academy of Sciences, U.S.A. Animals were obtained from the animal facility at the Research and Innovation Hub at Alamein International University.

#### Analysis of plasma samples

Separate volumes of 20 µL of rat plasma were transferred into a series of centrifuge tubes and spiked with different volumes of standard solutions of 5-FU (50 µg/mL), OXA (100 µg/mL), LV (100 µg/mL). To each tube ,16 µL of trimediazine (IS) solution (0.5 mg/mL) was added. Spiked plasma samples were then separately treated with 100 µL acetonitrile, vortex mixed for 5 min, then centrifuged at 4000 rpm for 10 min. The supernatants were separated into a set of clean tubes and the remaining residues were further washed with 1mL of acetonitrile, mixed then centrifuged. The supernatants and the washings were collected together, evaporated to dryness under vacuum, and the residues were then reconstituted with 100 µL of the diluting solvent. Reconstituted samples were chromatographed under the optimized "chromatographic conditions" after being filtered using 0.45 μm membrane filter.

#### Analysis of colonic media samples

Male Wistar rats weighing 200–250 g were utilized in the study obtained from the animal facility at the Research and Innovation Hub at Alamein International University. All the animals were kept in cages under standard laboratory conditions: a well-ventilated place, a regular 12 h day–night cycle, a controlled room temperature (25 ± 2 °C), and a relative humidity of 50 ± 10%. The rats had free access to tap water while diet was prohibited for 12 h before being scarified. They were acclimatized for 7 days to laboratory conditions before starting the experiments. Under thiopental anesthesia, the rats were sacrificed, and the caeci were exteriorized, legated at both ends, and cut loose. The collected caecal tissue was mixed and ground together. Volumes of 1 mL of the prepared caecal fluid were diluted to 20mL with acetonitrile from which 100 µL volumes were withdrawn and spiked with different volumes of standard solutions of the studied drugs along with the internal standard and the procedure of the sample preparation was carried out exactly as performed under the section of “Preparation of plasma samples”, Final analysis was performed exactly under the experimental section (Chromatographic conditions).

## Results and discussion

### Plan of the study

Since therapeutic protocols rely on diluting the investigated drugs using NS or D5W as universal diluents^38^, it is important to study the stability of drug mixtures diluted in NS/D5W in comparison with their corresponding single diluted solutions. It’s noteworthy to mention that the stability studies of 5-FU/LV admixtures revealed incompatibility between 5-FU and LV at high concentrations leading to precipitate formation with subsequent catheters blockage^23,24^. Moreover, the stability of 5-FU, OXA and LV ternary mixtures was previously studied in comparison to their binary combination 5-FU/OXA, 5-FU/LV and OXA/LV and their single drug administration. This report was concerned with investigating the stability of these drugs following dilution in buffer system of different pH values (5.0,6.0,7.5)^17^. Interestingly, it was found that in some cases the stability results of the drugs in their ternary mixture were significantly different from their binary or single solutions^17^. Thus, the plan of our work was based on the reported treatment protocol of the colonic cancer. This protocol specifies the use of Y- shaped infusion of OXA/LV for two hours followed by IV bolus of 5-FU and continuous IV infusion for 48 h^38^. The plan of our work relied on measuring the stability of binary admixtures of OXA/LV in comparison with their single solution along with single 5-FU solutions. Stability study of the cited drugs has been considered a matter of concern in previous studies which focused on single study of medications. Table S1 summarizes stability studies of the three cited drugs individually in NS/ D5W.

### Optimization of chromatographic conditions

Our preliminary trials were based on the chromatographic conditions used in the reported HPLC-DAD which relied on the separation of the three cited compounds using a gradient elution with mobile phase components of methanol / acidified water with trifluoroacetic acid all over a runtime of 68 min^17^.

Besides, other solvent systems were used for the determination of binary mixtures of 5-FU /LV^39^ and 5-FU /OXA^40^ composed of phosphate buffer with methanol as an organic modifier. In addition, single determination of each of the studied drugs was also reported, Table S2 summarizes mobile phase compositions used for the reported methods. Based on these previous reports^17,20,28–32,34,35,37,39–41^, it was thought of trying different mobile phase composition in an attempt to simultaneously determine the three cited drugs within a reasonable period of time.

HPLC-DAD is considered the *de facto* method for drug analysis. Compared with LC-MS/MS, the former is considered cheaper regarding instrumentation and maintenance. In addition, analysts can usually operate the system much easier than the LC-MS/MS which requires experience and high-level training of the operators. Moreover, the ability of DAD to assess peak purity based on absorbance measurements contributes to the selectivity of the method. It is also important to mention that the degree of sensitivity and selectivity provided by HPLC-DAD was considered satisfactory for the intended purpose of our study with no need to the sophisticated LC-MS/MS technique.

To optimize the chromatographic conditions, the effects of the pH of the aqueous phase, type of organic modifier, and methanol percentage were studied. Besides, selection of detection wavelengths of 5-FU, OXA and LV peaks was also studied.

#### Selection of detection wavelengths

DAD provides the advantage of multiple wavelengths selection, and each analyte could be monitored at its own wavelength of maximum absorbance to maximize the sensitivity. Accordingly, wavelengths of 266,254 and 288 nm were found optimum for the detection of 5-FU, OXA, and LV, respectively.

Additionally, DAD offers an easy and fast method for specificity checking by comparing absorption spectra that were recorded during peak elution at different points, and the peak purity was calculated and compared with threshold values.

#### Effect of pH of aqueous phase

Influence of the pH of the aqueous component of the mobile phase was studied by using aqueous phases at various pH values between 3 and 6 (adjusted using TFA 0.0001%, v/v). This was assessed under isocratic elution mode with a mobile phase combination (80% acidified water and 20% methanol). The pH had only a marked effect on the retention time of LV and nearly no effect on the retention times of 5- FU and OXA. Aqueous phases adjusted at pH 4.3 resulted in the proper separation of the studied compounds with the most stable baseline producing sharp symmetric peaks. Higher pH values led to increased retention of LV resulting in broad peaks.

#### Effect of type of organic modifier

Different organic modifiers (methanol, ethanol, and acetonitrile) were evaluated using mobile phases consisting of various percentages of the organic modifier, along with an aqueous phase of pH 4.3. Methanol was the best regarding resolution and shape of peaks. Ethanol showed too early elution of 5-FU and OXA was eluted as a forked peak which could not be improved by changing the percentage of ethanol in the mobile phase.

Acetonitrile could not achieve reasonable separation for three studied drugs even at low percentage in the mobile phase which resulted in an overlap between 5-FU and OXA peaks with poor-shaped LV peaks.

#### Effect of methanol percentage in the mobile phase

Methanol percentage effect was investigated under isocratic mode with mobile phase consisting of different percentage of methanol (6–22%) (Fig. 1b) and aqueous phase of acidified water, pH 4.3 (A).

Experimental studies revealed that methanol percentage of 16–22% resulted in incomplete baseline separation of 5-FU and OXA peaks. Decreased methanol concentration below 16% (6–16%) resulted in proper resolution between 5-FU and OXA, but late elution of broad LV peak. Decreased methanol concentration was accompanied with increased run time up to 40 min with 6% methanol (Fig. 1b).

Based on the above findings, a gradient elution mode (0 to 5 min, 86% V/V (A) was used, 5 to 10 min, 30% V/V (A) was used, 10 to 15 min, 86% V/V (A) was used and continued till 20 min analysis time) of varying percentage of methanol and acidified water (pH 4.3). Such system was selected for proper separation between the three cited drugs as sharp symmetric well-defined peaks with reasonable run time (less than 12 min, Fig. 1c).

### Method’s validation

The suggested HPLC procedure was validated in accordance with ICH guidelines^42^. The subsequent parameters were validated including linearity, limits of detection and quantitation, accuracy, precision, robustness, and selectivity. Regression calculations were derived using Microsoft Excel software, Microsoft Office 365. Stability data analysis was based on calculating the recovered drug concentration from the corresponding regression equation.

#### Linearity ranges and calibration graphs

Under the experimental conditions stated above (Chromatographic conditions), linear correlations were found by plotting peak areas for each compound against the corresponding concentrations in the ranges stated in Table 1. Results of the linear least squares regression treatment of analytical data were also provided including the slopes, intercepts, and correlation coefficients. High correlation coefficient values (r-values > 0.999) with minimal intercepts indicated high degree of linearity. Standard deviation of the slope (S_b_), the intercept (S_a_), and the residuals (S_y/x_) were also given. The degree of variation between the calculated and found (measured) y values was estimated from the calculated (S_y/x_). The closer the experimental points to the regression line, the smaller the (S_y/x_)values. An increase in the variance ratio (F values) for equal degrees of freedom indicates an increase in the mean of squares due to regression and decrease in the mean of squares due to residuals. High values for the (r) and (F) values are indicative of good regression lines^43^.

**Table 1:** Regression and statistical parameters for the determination of 5-Fluorouracil (5-FU), oxaliplatin (OXA), and leucovorin (LV) ternary mixtures using the proposed HPLC method (S_y/x_, standard deviation of residuals; S_a_, standard deviation of intercept; S_b_, standard deviation of slope; F, variance ratio, equals the mean of squares due to regression divided by the mean of squares about regression (due to residuals); LOD, limit of detection; LOQ, limit of quantitation).

Parameters	5-FU	OXA	LV
Detection wavelength (nm)	266	254	288
Linearity range (µg/mL)	0.125-50	0.3–125	0.2–100
Intercept (a)	20.003	1.213	-1.1228
Slope (b)	81.739	6.996	77.0586
Correlation coefficient (r)	0.9999	0.9999	0.9999
R ^2^	0.9999	0.9999	0.9999
s_a_	3.85	1.32	15.06
s_b_	0.21	0.02	0.32
S_y/x_	10.41	3.65	35.63
F	141051.7	73086.76	57052.76
Significance F	3.42417 × 10^− 20^	1.1793 × 10^− 20^	1.056 × 10^− 16^
LOQ (µg/mL)	0.12	0.3	0.2
LOD (µg/mL)	0.04	0.09	0.06

#### Detection and quantitation limits

The limit of detection (LOD) and limit of quantitation (LOQ) were calculated practically based on the analyte concentration with a signal-to-noise ratio of 3:1 for LOD and 10:1 for LOQ. The estimated LOD and LOQ values of the cited drugs were mentioned in Table 1 .The obtained low values of LOD and LOQ showed that the suggested HPLC method was appropriate for the determination of low concentrations^42^.

#### Accuracy

Accuracy of the suggested HPLC method was assessed by analyzing four laboratory-prepared mixtures of 5-FU, OXA, and LV at different ratios within the working concentration ranges (Table S3).It is noteworthy to mention that one of the analyzed mixtures was based on the concentration ratio used in actual treatment protocols ; (50 µg/mL) 5-FU: (3 µg/mL) OXA: (14.4 µg/mL) LV. Satisfactory recoveries with relative error < 2% indicated the high degree of accuracy of the proposed method.

#### Precision

Precision was assessed using three concentration levels within the linearity range of each compound: low, medium, and high. The analysis was performed on the same day for evaluating the intra-day precision, *n* = 3, and on three successive days for inter-day precision, *n* = 9. The percentage relative standard deviation (RSD%) was calculated and small values less than 2% were obtained showing a high degree of the precision of the proposed method (Table S4).

#### Robustness

The robustness of the proposed method was tested by making slight deliberate changes in the experimental conditions such as pH of the acidified water in the mobile phase (± 0.2 pH units) and the detection wavelength (± 2 nm). There were no significant alterations in the peak areas or the retention times of the cited compounds, with RSD% of less than 2% indicating a high degree of method’s robustness (Table S5).

#### Selectivity

Selectivity refers to an analytical method’s ability to distinguish and quantify analytes in the presence of other components in a sample. DAD was also beneficial for checking absorption spectra of peaks and ensuring peaks’ purity when the calculated purity indices didn’t exceed the threshold values.

### System suitability testing

The chromatographic system’s suitability parameters were evaluated as part of the analytical procedure to ensure the operating system’s efficacy. Method performance data was listed in Table S6. Good peaks’ separation was indicated from high resolution values (R_S_ > 1.5). Also, the high number of theoretical plates revealed good column efficiency. Furthermore, the tailing factor was used to assess the degree of peak’s symmetry and the results were within the acceptable range (T < 2), indicating a suitable level of symmetry of peaks^44^. Figure 1c represents a typical chromatogram of the studied drugs at concentrations mimicing those used in actual treatment protocol. Also, a typical chromatogram of a standard mixture of the cited drugs with their corresponding absorption spectra and peak purity graphs is demonstrated in .S1.

### Stability study

The stability of the three studied drugs was investigated at two temperature conditions (room temperature 25 ^o^C, refrigeration 2–8 ^o^C) in two different diluent solutions (NS and D5W). In each case, the physical and the chemical stability of the drugs were followed up over approximately two weeks. Chemical analyses were performed based on the procedures (Chromatographic conditions) and timeline (Stability assay and sampling timeline) outlined in their experimental section. It was observed that no physical degradation signs marked as changes in color, production of precipitate or turbidity creation was observed in all cases.

#### Stability study in D5W solution

##### Room temperature (RT) 25 ^o^C

Table 2 demonstrates the stability results over the period of study. Binary mixtures of OXA and LV showed some chemical degradation as follows; OXA rapidly degraded to 61.5% of its initial concentration on day 2 with a further drop to 40% by day 4 which continued to drop below its detection limit on day 16. However, LV showed a slower rate of degradation compared with OXA as it declined to 93.8% on day 2 and down to 70.5% at the end of the study, day 16. On the contrary, single solutions of OXA and LV had a more stable profile than the case of their corresponding binary mixtures where both drugs showed nearly no degradation until day 4 for OXA and day 7 for LV. Additionally, only slight degradation of both drugs, lower than 5%, was recorded after 16 days. The study also revealed the chemical stability of 5-FU with only minor degradation of 2% over 16 days.

**Table 2:** Stability data of samples of the studied compounds dispensed in 5% dextrose solution kept at room temperature (RT) /refrigeration (2–8 ^o^C) for 16 days all calculated concentrations were based on final diluted solutions injected in the HPLC system as follows: 50 µg/ml (5-FU), 3 µg/ml (OXA), and 14.40 µg/ml (LV) corresponding to original concentration of 5000 µg/ml (5-FU), 300 µg/ml (OXA), and 1440 µg/ml (LV) in infusion bags(ND: not detected). 90–95% of drugs’ initial concentrations with no signs of physical change shows drugs’ stability.

(% Intact drug), Concentration* (µg/mL)								
	Day 0	Day 2	Day 4	Day 7	Day 9	Day 11	Day 14	Day 16
Oxaliplatin								
Oxaliplatin (single/RT)	100.0 3.00	100.0 3.00	100.0 3.00	98.9 2.96	98.6 2.95	98.3 2.94	96.7 2.90	96.7 2.90
Oxaliplatin (single/2-8^O^C)	100.0 3.00	100.0 3.00	99.9 2.99	98.8 2.96	98.4 2.95	98.1 2.94	95.6 2.86	95.6 2.86
Oxaliplatin (binary/RT)	100.0 3.00	61.5 1.84	40.9 1.22	37.4 1.12	25.6 0.76	22.7 0.68	12.0 0.36	ND
Oxaliplatin (binary/2-8^O^C)	100.0 3.00	86.8 2.60	75.3 2.25	67.0 2.01	64.0 1.91	60.9 1.82	49.7 1.49	49.7 1.49
Leucovorin								
Leucovorin(single/RT)	100.0 14.40	100.0 14.40	100.0 14.40	100.0 14.40	100.0 14.39	98.3 14.15	97.5 14.04	97.5 14.04
Leucovorin (single/2-8^O^C)	100.0 14.40	100.0 14.40	100.0 14.40	100.0 14.40	100.0 14.40	100.0 14.40	100.0 14.40	100.0 14.40
Leucovorin (binary/RT)	100.0 14.40	93.8 13.51	86.2 12.41	81.5 11.73	77.1 11.10	71.8 10.34	70.5 10.15	70.5 10.15
Leucovorin (binary/2-8^O^C)	100.0 14.40	100.0 14.40	98.8 14.23	97.1 13.98	96.9 13.95	95.1 13.70	90.5 13.02	90.5 13.02
5-FU								
5-FU (single/RT)	100.0 50.00	100.0 50.00	100.0 50.00	100.0 50.00	100.0 50.00	98.5 49.23	98.0 49.00	98.0 49.00
5-FU (single/2-8^O^C)	100.0 50.00	100.0 50.00	100.0 50.00	100.0 50.00	100.0 50.00	100.0 50.00	100.0 50.00	100.0 50.00

##### Refrigeration (2–8 ^o^C)

**Fig. 2:**
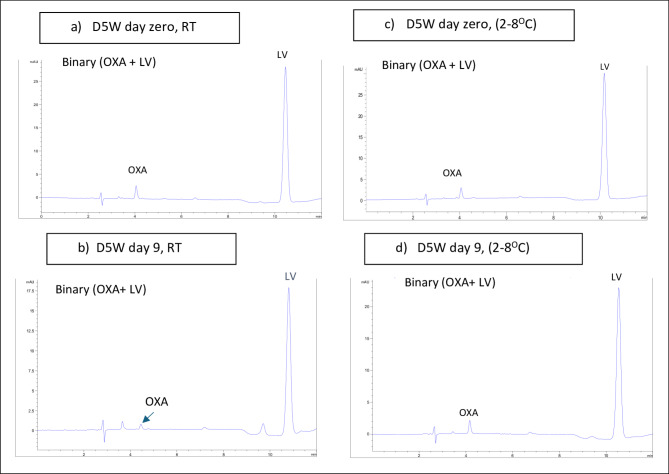
Typical chromatograms of samples of a binary mixture of Oxaliplatin (OXA) (3 µg/mL) and Leucovorin (LV) (14.4 µg/mL) dispensed in 5% dextrose solution at (**a**) 0-time stored at room temperature (RT), (**b**) day 9 stored at room temperature (RT), (**c**) 0-time stored at 2-8^O^C and (**d**) day 9 stored at 2-8^O^C, all measured at 254 nm.

**Fig. 3:**
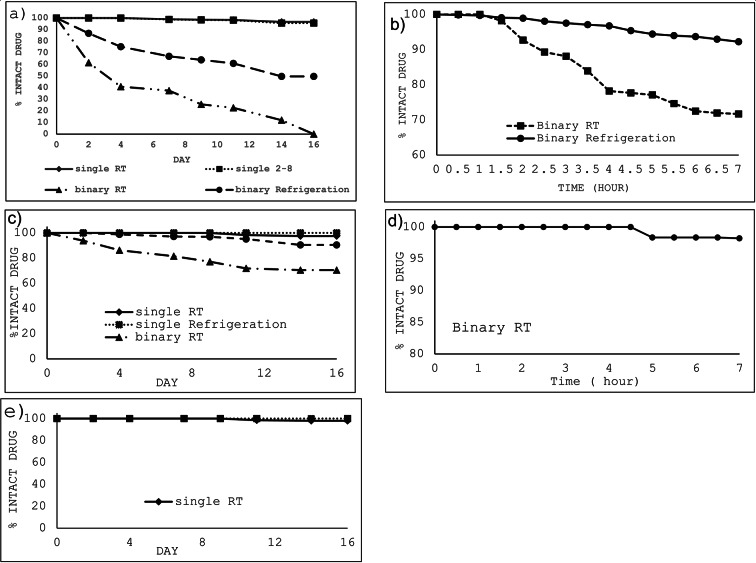
Percentage of concentration versus time profile of (**a**) OXA in D5W either separately or in a binary mixture with LV over 16 days, (**b**) OXA in D5W either separately or in a binary mixture with LV over 7 h, (**c**) LV in D5W either separately or in a binary mixture with OXA over 16 days, (d) LV in D5W in a binary mixture with OXA over 7 h, (**e**) 5-FU in D5W over 16 days. All solutions were investigated at room temperature (RT) and refrigerated at 2–8 ^o^C.

Refrigerated binary mixtures of OXA and LV were found to be more stable compared with those stored at room temperature since their rate of decline in concentration were slower; concentration of OXA declined to 86.8% by day 2 and to 49.7% at the end of the study, while concentrations of LV remained above 90% until the 16-day. Also, single OXA solution showed no significant drug loss for 16 days as its concentration was higher than 95.6%. No concentration decrease was detected in cases of single 5-FU and single LV solutions (Table 2). Figure 2 shows representative examples of the chromatograms of D5W-diluted infusion solutions kept for 9 days compared with day 0. Additional chromatograms show the stability of the drugs on study’s days are also provided in the supplementary section (Fig.S2). Stability results of D5W solution were clarified in Fig. 3.

Detailed investigation of the stability of solutions showing marked degradation on day 2 was rechecked every 30 min for 7 h. As shown in Fig. 3b, OXA stability rapidly declined when mixed with LV and keeping the mixture at room temperature. The solution remains stable (> 90%) for only 2 h above which significant degradation occurs. On the contrary, Fig. 3c shows considerable stability of LV in its binary mixture with OXA for more than 7 h (Table S7).

#### Stability study in NS solution

##### Room temperature (RT) 25 ^o^C

The combination of OXA and LV ensured a steady LV concentration over the first two days with slight degradation to 95.3% by day 5 and to 90% over the 15 days of study. OXA exhibited a fast decrement pattern in NS solution at RT; 41% degradation after only 2 days with continuous decrease in its concentration to less than its limit of quantitation by day 7 and less than its limit of detection afterword.

The same was found for single OXA solution diluted in NS and kept in room temperature as noticed with its binary mixture. A rapid concentration decrease by 39% was recorded over 2 days with excessive decrease in its concentration below its limit of quantitation or limit of detection on days 9 and 12, respectively. Regarding single solutions of either LV or 5-FU, no significant degradation was found, only 1% decrease in LV concentration and 1.5% decrease in 5-FU concentration were observed at the end of the study (Table 3).

**Table 3:** Stability data of samples of the studied compounds dispensed in normal saline solution kept at room temperature (RT) /refrigeration (2–8 ^o^C) for 15 days all calculated concentrations were based on final diluted solutions injected in the HPLC system as follows: 50 µg/ml (5-FU), 3 µg/ml (OXA), and 14.40 µg/ml (LV) corresponding to original concentration of 5000 µg/ml (5-FU), 300 µg/ml (OXA), and 1440 µg/ml (LV) in infusion bags (NQ: not quantified, ND: not detected). 90–95% of drugs’ initial concentrations with no signs of physical change shows drugs’ stability.

(% intact drug), concentration* (µg/mL)								
	Day 0	Day 2	Day 4	Day 7	Day 9	Day 11	Day 14	Day 15
Oxaliplatin								
Oxaliplatin (single/RT)	100.0 3.00	61.0 1.83	27.7 0.83	15.3 0.45	NQ	ND	ND	ND
Oxaliplatin (single/2-8^O^C)	100.0 3.00	87.2 2.61	80.1 2.40	78.2 2.34	75.0 2.25	56.0 1.68	51.6 1.54	51.5 1.54
Oxaliplatin (binary/RT)	100.0 3.00	59.0 1.77	15.1 0.45	NQ	ND	ND	ND	ND
Oxaliplatin (binary/2-8^O^C)	100.0 3.00	85.0 2.55	69.4 2.08	41.7 1.25	39.9 1.19	34.8 1.04	30.0 0.90	30.0 0.90
Leucovorin								
Leucovorin (single/RT)	100.0 14.40	100.0 14.40	100.0 14.40	100.0 14.40	100.0 14.40	100.0 14.40	99.1 14.27	99.0 14.25
Leucovorin (single/2-8^O^C)	100.0 14.40	100.0 14.40	100.0 14.40	100.0 14.40	100.0 14.40	100.0 14.40	100.0 14.40	100.0 14.40
Leucovorin (binary/RT)	100.0 14.40	100.0 14.40	95.3 13.73	93.5 13.46	90.7 13.06	90.4 13.02	90.2 12.98	90.1 12.97
Leucovorin (binary/2-8^O^C)	100.0 14.40	100.0 14.40	100.0 14.40	99.8 14.36	98.8 14.22	98.4 14.17	98.3 14.14	98.2 14.14
5-FU								
5-FU (single/RT)	100.0 50.00	100.0 50.00	100.0 50.00	99.0 49.50	98.9 49.45	98.9 49.44	98.5 49.25	98.5 49.22
5-FU (single/2-8^O^C)	100.0 50.00	100.0 50.00	100.0 50.00	100.0 50.00	100.0 50.00	99.9 49.95	99.8 49.90	99.8 49.89

##### Refrigeration (2–8 ^o^C)

Binary mixture solutions of OXA and LV kept at 2–8 ^o^C recorded significant improvement in the stability of both drugs over room temperature despite that a decrease in OXA concentration was recorded as it dropped to 85% ( 15% degradation ) over 2 days and to 30% over 15 days while a slight decrease by 2% in LV concentration was noted at the end of the study.

Single solution of OXA showed a noticeable lesser concentration decrease profile compared to its solution at room temperature since it decreased to 87.2% after the first 2 days and 51.5% over the study. This reported stability behavior of OXA in NS was in accordance with literature indicating that chloride ions resulting in noticeable degradation of OXA solutions^17,20^. Single solutions of either LV or 5-FU showed nearly no significant degradation till the end of the study (Table 3).

**Fig. 4:**
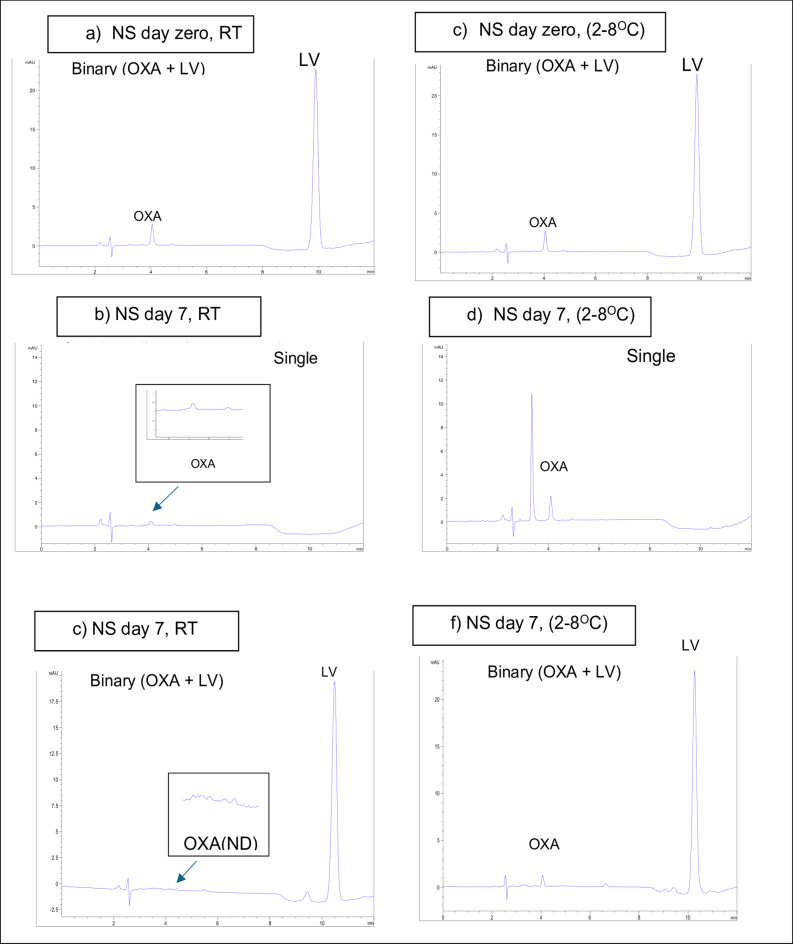
Typical chromatograms of samples in NS solution (**a**) binary mixture of OXA (3 µg/mL) and LV (14.4 µg/mL) at 0 time/RT, (**b**) OXA (3 µg/mL) at day 7/RT, (**c**) binary mixture of OXA (3 µg/mL) and LV (14.4 µg/mL) at day 7/ RT (**d**) binary mixture of OXA (3 µg/mL) and LV (14.4 µg/mL) at 0 time/ 2-8^O^ C, (**e**) OXA (3 µg/mL) at day 7/ 2-8^O^ C, (**f**) binary mixture of OXA (3 µg/mL) and LV (14.4 µg/mL) at day 7/ 2-8^O^ C. All solutions were measured at 254 nm. **ND stands for not detected.

**Fig. 5:**
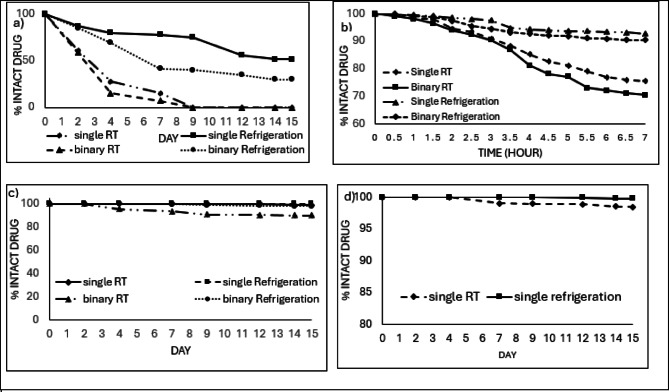
Percentage of concentration versus time profile of (**a**) OXA in NS either separately or in a binary mixture with LV over 15 days, (**b**) OXA in NS either separately or in a binary mixture with LV over 7 h, (**c**) LV in NS either separately or in a binary mixture with OXA over 15 days, (**d**) 5-FU in NS over 15 days. All solutions were investigated at room temperature (RT) and at refrigerated at 2–8 ^o^C.

Representative examples of typical chromatograms of samples of the studied compounds dispensed in NS for 7 days as compared with day 0 were given in Fig. 4. All chromatographs showing the stability on different days of the study were shown in Fig.S3, supplementary section. Figure 5 represents a plot of % intact drug over the studied period.

As per the D5W solutions, the stability of OXA in NS was further studied stepwise every 30 min for 7 h (Fig. 5b ). The results revealed the stability of refrigerated OXA solutions in NS for up to 7 h for both single and binary mixtures. However, single or binary OXA solutions were found stable when kept at room temperature for only 3 h. In this respect, it was shown that the stability behavior of OXA in NS in its binary mixtures didn’t differ significantly from its single solutions when kept at the same temperature (Table S7).

In all cases peak purity was checked by DAD to ensure the purity of each peak with no co-elution of any degraded products under the studied temperature conditions.

### Analysis of biological samples

#### Analysis of spiked rat plasma samples

The applicability of the proposed HPLC method was extended to the simultaneous determination of 5-FU, OXA, and LV in rat plasma samples spiked with the studied compounds. Depending on the dosage and drug administration regimen prescribed^38^, the amounts of these compounds in plasma samples of cancerous patients show a great variation^12,45–49^.The maximum concentrations (C_max_) of 5-FU, OXA, and LV were reported within the following concentration ranges: 10–125, 1.52-5, and 3–10.89 µg/mL, respectively .Our study was based on the treatment protocol using the three drugs for the management of colon cancer using the ratio of 50:3:14 for 5-FU: OXA: LV^38^. Accordingly, C_max_ of 15 µg/mL 5-FU, 5 µg/mL OXA, and 3.7 µg/mL LV were selected in the study. Moreover, concentrations below the C_max_ values were also spiked in rat plasma to emphasize the potential of the proposed method to analyze low concentrations.

It is noteworthy to mention that analysis of biological samples was based on the internal standard (IS) method where the peak area ratios of the studied drugs were related to their concentrations. Trimediazine was selected as the working internal standard since it produced a sharp peak with no interference with the analytes’ peaks. A concentration of 80 µg/mL was chosen since it gave a reasonable response compared to the three spiked drugs in plasma. Matrix-based calibration was obtained by plotting the concentration of drugs recovered from plasma samples spiked with standard drugs along with internal standard, trimediazine, against their concentrations (Table S8).

**Fig. 6:**
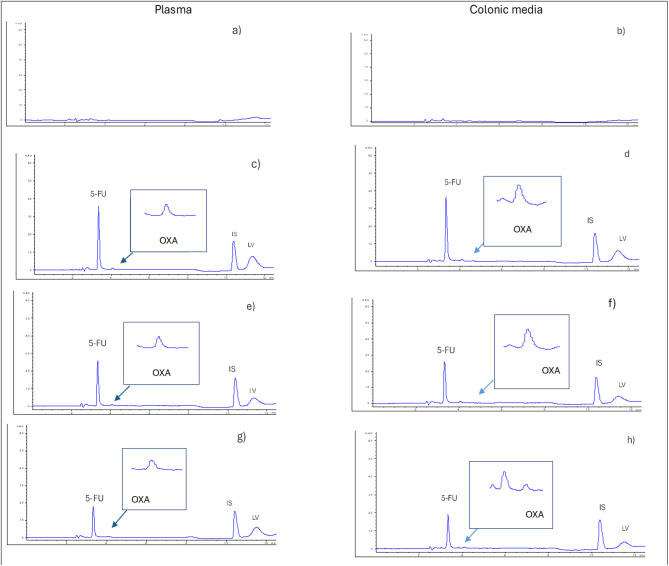
Typical chromatograms of (**a**) blank plasma, (**b**) blank colonic media sample, (**c**) sample of IS (80 µg/mL), 5-FU (3 µg/mL), OXA (1 µg/mL) and LV (0.74 µg/mL) In spiked plasma, (**d**) sample of IS (80 µg/mL), 5-FU (3 µg/mL), OXA (1 µg/mL) and LV (0.74 µg/mL) In spiked colonic media, (**e**) sample of IS (80 µg/mL), 5-FU (2.1 µg/mL), OXA (0.7 µg/mL) and LV (0.52 µg/mL) in spiked plasma, (**f**) sample of IS (80 µg/mL), 5-FU (2.1 µg/mL), OXA (0.7 µg/mL) and LV (0.52 µg/mL) in spiked colonic media, (**g**) sample of IS (80 µg/mL), 5-FU (1.5 µg/mL), OXA (0.5 µg/mL) and LV (0.37 µg/mL) in spiked plasma and (**h**) sample of IS (80 µg/mL), 5-FU (2.1 µg/mL), OXA (0.7 µg/mL) and LV (0.52 µg/mL) in spiked colonic media. All chromatograms were measure using optimized chromatographic conditions measured at 266 nm.

Protein precipitation was applied as an easy and efficient sample clean up way. Acetonitrile was used as an organic precipitant and extracting solvent since it produced a good recovery for the three analyzed drugs in addition to a small baseline noise level. To avoid excessive dilution, treated samples were evaporated to dryness then the obtained residues were reconstituted with the diluting solvent of water/methanol (90/10, v/v). Recovery was evaluated by using rat plasma samples spiked with different concentrations of the three cited drugs and chromatographed as mentioned in the experimental section (Chromatographic conditions). In this respect, three different mixtures were prepared as follows: 3 µg/mL 5-FU, 1 µg/mL OXA, and 0.74 µg/mL LV, (mixture 1), 2.1 µg/mL 5-FU, 0.7 µg/mL OXA, and 0.52 µg/mL LV, ( mixture 2), besides 1.5 µg/mL 5-FU, 0.5 µg/mL OXA, and 0.37 µg/mL LV (mixtures 3). Recovery % and E_*r*_ % values indicated appropriate application of sample treatment method (Table 4). Figure 6 shows typical chromatograms of the spiked plasma samples compared with blank plasma samples.

**Table 4:** Results of analysis of rat plasma samples and colonic media samples spiked with the studied drugs using the proposed HPLC method for the determination of 5-Fluorouracil (5-FU), oxaliplatin (OXA), and leucovorin (LV).

Nominal value, (µg/mL)			Plasma samples						Colonic media samples					
			Recovery, % ± SD			E_*r*_ %			Recovery, % ± SD			E_*r*_ %		
5-FU	OXA	LV	5-FU	OXA	LV	5-FU	OXA	LV	5-FU	OXA	LV	5-FU	OXA	LV
3	1	0.74	98.7 ± 0.02	99.2 ± 0.01	98.6 ± 0.01	− 1.3	− 0.8	− 1.4	99.1 ± 0.01	99.0 ± 0.009	99.1 ± 0.01	-0.9	− 1	− 0.9
2.1	0.7	0.52	99.0 ± 0.01	98.8 ± 0.009	99.1 ± 0.01	− 1.0	-1.2	− 0.9	98.5 ± 0.03	98.9 ± 0.01	99.0 ± 0.009	-1.5	− 1.1	− 1
1.5	0.5	0.37	98.5 ± 0.02	99.0 ± 0.002	99.2 ± 0.005	− 1.5	− 1	− 0.8	99.3 ± 0.02	99.1 ± 0.008	98.8 ± 0.004	-0.7	− 0.9	− 1.2

The specificity of the method was assessed by analyzing blank plasma samples (following protein precipitation) along with plasma samples spiked with the cited drug at their LOQ concentration levels. Since no interfering peaks in plasma samples were detected at the analytes’ retention times, it was concluded that no endogenous plasma components could have affected the assay. Peak purity was also assessed by purity indices values being less than threshold values, as calculated by DAD.

#### Analysis of colonic media sample

Since our study focused on the therapeutic protocol intended for the treatment of the colon cancer, it was thought to extend the applicability of the proposed HPLC-DAD to the determination of colonic media samples spiked with the cited drugs. Concentration levels used for spiking the colonic samples were the same as those used for spiking plasma samples, along with Trimediazine (IS) (Preparation of infusions). Sample treatment was exactly as carried out for plasma sample. Also, the method’s specificity was assessed exactly as mentioned before under “Analysis of spiked rat plasma samples”. Treated mixtures were analyzed using the optimized HPLC-DAD conditions mentioned under the experimental section (Chromatographic conditions). Matrix-based calibration was obtained by the same method shown in "Preparation of infusions" (Table S9), good recovery values showed the effectiveness of the proposed sample treatment method (Table 4). Typical chromatograms of colonic media samples spiked with the three studied drugs along with blank colon sample (Fig. 6). Additional chromatograms are seen in Fig. S4.

### Comparison with the reported methods

To our knowledge, only one report was found in the literature dealing with the simultaneous determination of 5-FU, OXA and LV^17^. This work was concerned with studying the stability of the three drugs in acetate buffer of different pH values 5, 6, and 7.5. However, the applicability of the proposed HPLC-DAD method developed in our work was extended to stability studies of the cited compounds in NS and D5W studied at different temperatures; room temperature (25^o^C) and refrigeration (2-8^o^C). The analytical performance of our proposed method was compared with the reported one^17^, it was found that values of LOD and LOQ of OXA and LV obtained by the current study were lower than those obtained by the reported method. This improved high detectability enabled the determination of these drugs at low concentrations. In addition, the runtime of the proposed method (20 min ) is much lower than that of the reported one (> 68 min )^17^, about one third. This is followed by increasing of throughput of the method along with reducing the waste and the material used with their negative impact on the environment besides reducing energy consumption which all contribute to the method’s greenness.

### Greenness, blueness and whiteness evaluation of the proposed HPLC method as compared with the reported HPLC method

#### Greenness evaluation

Environmental impact has been considered as one of the most crucial factors when choosing analytical methodology. Eco friendliness is dependent upon the type of chemicals and solvents used, as well as the generated waste. In this regard, a number of matrices have been authorized for examining the degree of greenness and environmental friendliness of analytical techniques. They include analytical NEMI, ESA, GAPI, and AGREE^50–54^.

The first method utilized to assess the level of environmental friendliness of the proposed HPLC-DAD method was NEMI where the greenness profile of a four-field pictogram was employed, each quadrant was left blank if the designated requirement was not met and is colored green otherwise. The four requirements were as follows: the substance must not be hazardous, corrosive, persistent, bio accumulative, or toxic (PBT), nor should it produce a significant quantity of waste (more than 50 g per sample analysis)^54–56^. Since the solvent system was neither corrosive nor PBT, the waste generated per sample was less than 50 g, and the solvents utilized in both methods were of considerable hazards (methanol and TEA), the NEMI pictograms of the published and proposed HPLC methods showed three green quadrants and the quadrant depicting the method’s hazardous potential was left blank (Table 5).

The second tool used to assess the greenness was ESA. This method depends on computing the overall penalty points according to the type of reagents, which include all solvents used, as well as the instrument used in terms of energy consumption, occupational hazards, and waste generated during the analysis^56^. Comparing the ESA scores between the proposed and the reported HPLC-DAD methods, both methods obtained the same ESA score of 84 since they both used the same solvent system ; methanol / acidified water adjusted with TFA and generated waste more than 10 mL (Table 5). However, the two methods were considered acceptably green. Although Eco-Scale has numerous benefits, including ease of use, semi-quantitative computation of chemical and waste amounts, quantitative information regarding the environmental effects of analytical methods, and evaluation of various environmental impact aspects. One of its shortcomings, though, is that there aren’t any more quantifiers that can distinguish between method applications on a micro- and macro-scale. Furthermore, in the event of a negative environmental effect, the outcome is not instructive, and as a consequence, it does not support the method’s improvement throughout the design phase in this regard^56,57^.

Additionally, we employed the more recent tools, GAPI, which was first used in 2018^58^ and AGREE, which was just adopted in 2020^59^, to assess the method’s greenness. The GAPI tool utilizes colorful, fifteen-zoned pentagrams to reflect the greenness of the created analytical methods. The resulting GAPI pictograms showed the two methods’ high level of environmental friendliness since only three red, four yellow and eight green zones were recorded in each method. The red zones represented the usage of non-green solvents (zone 7), waste volumes greater than 10 mL(zone 14), and inadequate waste treatment (zone 15). The four yellow zones stood for the following parameters : using simple processes such as extraction and filtration (zone 5); a solvent that was moderately hazardous (zone 10); a solvent that had a flammability rating of two or three (zone 11); and energy consumption less than 1.5 kWh per sample (zone 12).Finally, the remaining 8 zones were colored green corresponding to the following: inline collection (zone 1), no preservative (zone 2), no transport (zone 3), no storage (zone 4), no extraction (zone 6), no advanced treatment like derivatization (zone 8), sample volume < 10 (zone 9), and hermetic system use (zone 13)^54,58^.

Conversely, AGREE offers a colorful pictogram with a central circle with a number value and 12 surrounding sectors. The 12 areas display adherence to the 12 green analytical chemistry (GAC) principles in various colors. The closer the central numerical score is to unity; the greener the analytical method is^54,59^. Regarding the AGREE pictogram, it is noteworthy to mention that the proposed method had a better overall green score (0.76) than the reported method^17^ (0.70). The greenness evaluation with the descriptive AGREE tool clearly validates better the proposed method’s eco-friendliness, which could not be measured with the other assessment techniques. This might be ascribed to the superiority of the most recent AGREE tool, when compared to ESA, NEMI, and GAPI, because the former incorporates the full assessment of the 12 GAC principles. For the proposed method, no section in the perimeter was red, while four sections were colored yellow owing to generation of a large volume of analytical waste (Sector 7), sample throughput was 3 sample / hour (Sector 8), being LC technique with high energy consumption (Sector 9), and the biological origin of some of the reagents used (Sector 10). The remaining eight sections of the pictogram were green for the obedience of the following criteria of GAC, online analysis (Sector 1), a small amount of sample of 0.02 mL (Sector 2), the inline position of the device (Section 3), few steps of sample preparation (Sector 4), automation of the applied method with minimum sample preparation (Sector 5), absence of derivatization (Sector 6), degree of toxicity (Sector 11) and the degree of flammability and corrosiveness of the reagents used (Section 12). Nearly the distribution of colored sections was the same in the pictograms of both methods except for sectors 7, 8, and 9, since the volume of generated waste was approximately double than that in the proposed method (Sector 7), very long runtime which led to sample throughput 0.88 sample / hour (Sector 8) and runtime also affected energy consumption (Sector 9) so these sections were colored dark orange in the reported method^17^ while yellow in the proposed method. The differences in the chromatographic conditions regarding flow rate, and runtime with subsequent waste generation resulted in the relatively superior AGREE score for the proposed method (Table 5). In conclusion, greenness evaluation tools such as ESA, GAPI, and AGREE evaluated the proposed method as excellent green method.

#### Blueness evaluation

Comparing to green assessment tools such as NEMI, ESA, GAPI, and AGREE which concentrate on evaluating environmental impact and greenness, Blue Applicability Grade Index (BAGI)^60,61^ quantitatively evaluates the feasibility and applicability of analytical techniques for applied analysis. BAGI allows for a comprehensive evaluation of an analytical method’s blueness by considering 10 key factors: type of analysis, number of analytes, type of analytical technique, sample throughput, needs of sample preparation, numbers of samples /h, required reagents/materials, preconcentration requirement, degree of automation, and amount of sample. Each of the ten variables was rated on a scale of 2.5 (worst) to 10 (best) where the score of 10, 7.5, 5.0, and 2.5 points correspond to dark blue, blue, light blue, and white, respectively. The composite BAGI score is the geometric mean of the 10 individual criterion scores. A higher BAGI score points to an analytical approach that is more relevant, applicable, and fit-for-purpose. BAGI was applied to evaluate and compare the blueness of our proposed HPLC-DAD method to the reported one^17^. Our method had a score of 90 while the reported method^17^obtained a score of 87.5. The high BAGI score of both methods reflected excellent blueness since they achieved the best score (dark blue color) in 7 factors and blue color in two factors: number of analytes and type of analytical technique. The factor corresponding to the number of samples /h led to difference between the two methods as the proposed method got 5 ( light blue ) but the reported one^17^obtained the worst score (white) due to its very long runtime (Table 6). It is important to mention that BAGI relies on using geometric means rather than arithmetic means since the former is more sensitive to data extremes and is appropriate for the multiplicative character of the criterion with a greater relative weight for low scores. Although BAGI examines real-world applicability, it does not give a comprehensive assessment of sustainability. To analyze analytical value, greenness, and practicality, the RGB12 algorithm was also used.

**Table 5:**
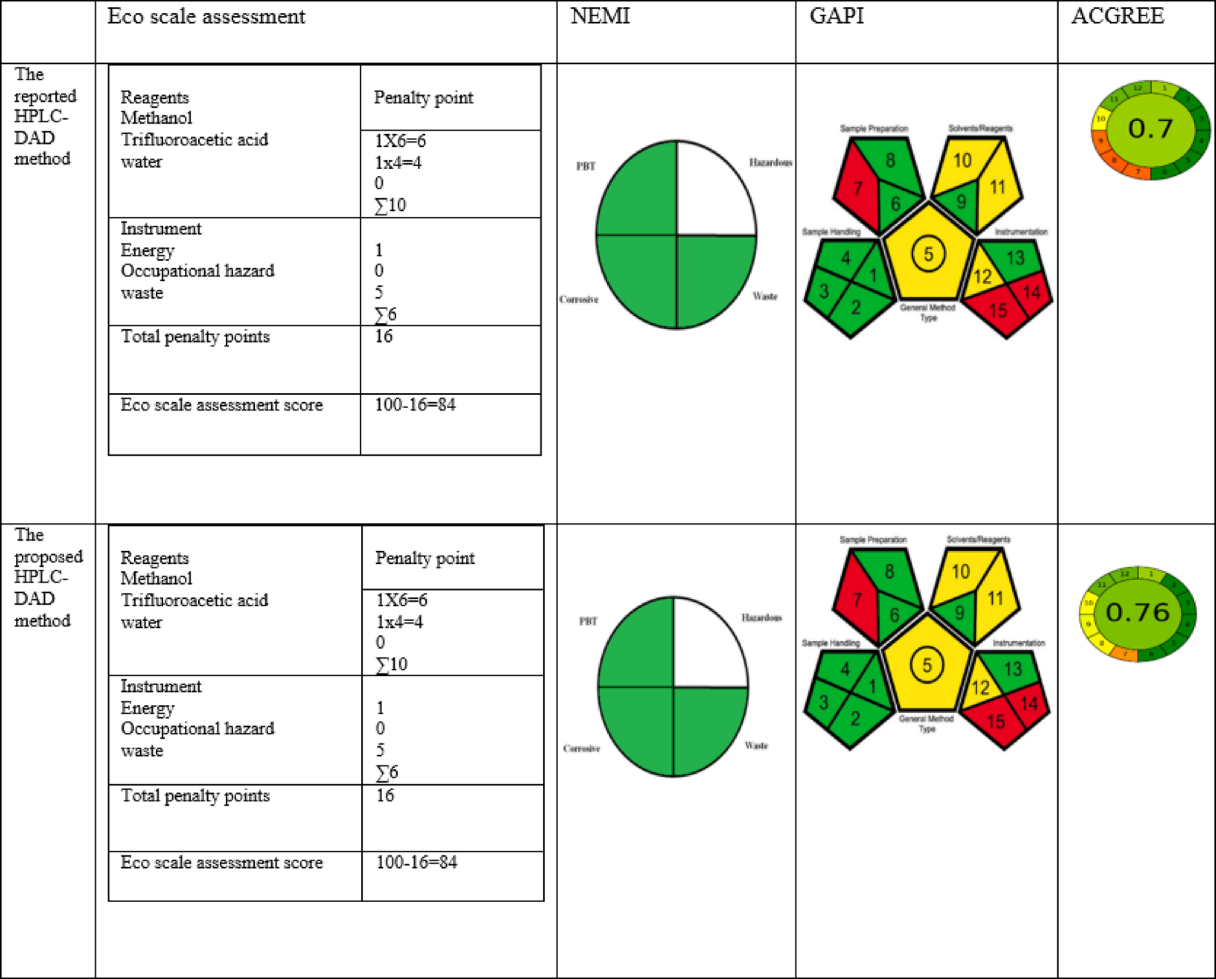
Evaluation of the degree of greenness of the proposed HPLC-DAD method (current study) in comparison with the reported HPLC-DAD method^17^ using Eco scale assessment ,NEMI, GAPI, and AGREE assessment tools.

#### Whiteness assessment

The broad term “sustainable development, SD” has recently been introduced into the scientific research community^52,53,62,63^. SD of analytical methods combines three pillars: social demands, validation criteria compliance, and eco-friendliness and greenness aspects, in addition to practical and economical aspects. It is important to note that greenness assessment tools, such as NEMI, ESA, GAPI, and AGREE, only assess the environmental impact of analytical methodologies; they do not guarantee the method’s sustainability because they do not provide a comprehensive picture of the method’s applicability or economic issues. To this end, the Red-Green-Blue (RGB12) model is a comprehensive evaluation tool that evaluates analytical performance (red), GAC compliance (green), and ultimately economic and productivity elements (blue). The evaluation tool’s impartiality and flexibility are enhanced using an online Excel sheet and a unique algorithm to give color ratings to each of the three basic criteria. The proposed HPLC method’s level of whiteness was assessed using the RGB 12 model in comparison with the reported HPLC method^17^. The whiteness score of the proposed one was 96.3% while the reported method’s score was 79.6%. The long runtime of the reported method^17^ resulted in decreasing the percentage of both greenness and blueness parameters. The large amount of generated waste and consumed energy resulted in a decrease in G% and decline in cost and time efficiency, due to elongated runtime of > 68 min, caused the decrease in B%. In addition, the lower levels of LOD and LOQ reported for determination of OXA and LV in the proposed method contributed to the improved analytical performance with the higher % of redness ,100% for our method compared with 93.8% for the reported method^17^. This highlights that the proposed methodology’s overall sustainability is superior compared to the reported one^17^ regarding the whole three aspects, analytical performance (R), greenness(G) and validation to its cost and applicability (B) (Table 6) .

**Table 6:**
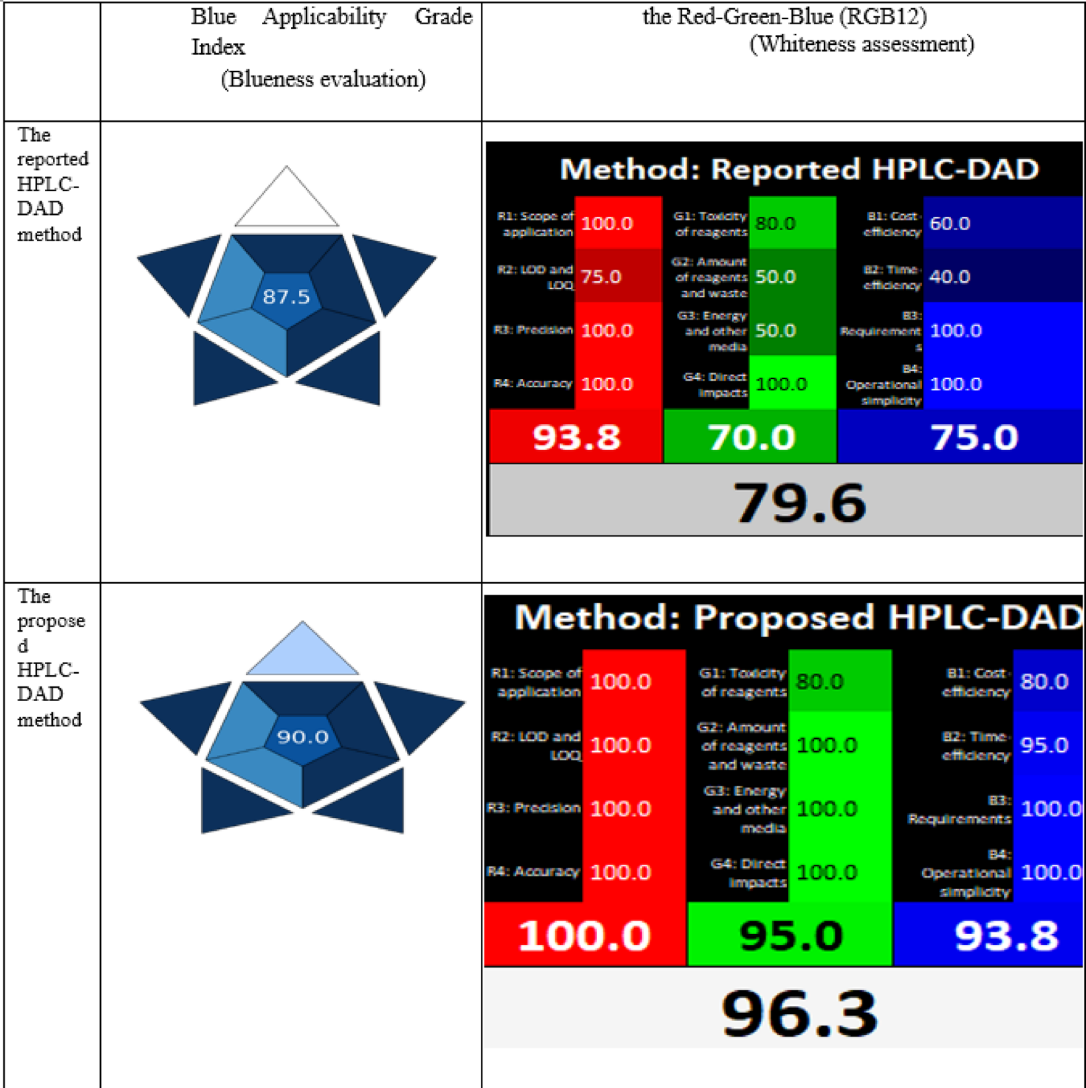
Evaluation of the degree of blueness and whiteness of the proposed HPLC-DAD method (current study) in comparison with the reported HPLC-DAD method^17^.

## Conclusion

An HPLC-DAD method was developed and validated for the stability studies of FOLFOX in the infusion bags. Since therapeutic protocols rely on diluting medications using NS or D5W as universal diluents and since the drug diluents could play an important role on the stability of drugs, stability studies were extended to mixtures’ solutions diluted in NS/ D5W in comparison with their corresponding single diluted solutions. Two temperature settings were examined, room temperature (25^o^C), and refrigeration (2–8 ^o^C). The chemical nature and the incompatibility studies of FOLFOX ingredients; OXA, LV ,5-FU, necessitates a sequence of the administration as follows: Y- shaped tube containing OXA and LV in each branch followed by IV bolus of 5-FU. This study focuses on the investigation of the stability of binary combination of OXA/LV, being found in the junction tubing in actual administration set, as compared with their single solution, as found in each branch of the Y-shaped tube. The results showed that single solutions of each drug (OXA/LV) suspended in D5W were stable all over the period of study at both temperatures. On the contrary, their binary mixture was stable for only 2 h for OXA at room temperature. These findings revealed the safe storage of single OXA/LV diluted in D5W solution, enabling the possibility of their early preparation in clinical practice with no need for on- time setting which may be a burden in some cases.

Regarding NS solution, the instability of OXA in NS at room temperature forced the necessity of its avoidance as a diluent in the administration set despite the stability of LV in NS. On the other hand, 5-FU could be safely dispensed in either diluent. It is also important to mention that treatment protocols depending on using a binary anticancer combination of 5-FU and LV, being taken separately, could also be diluted in NS or D5W solutions without any degradation. The use of NS in drugs’ dispensing resulting in preventing the use of D5W which should be avoided in some clinical situations since it is reported to produce hyperglycemia, phlebitis with severe venous pain to some patients.

The proposed method could also be successively applied to the determination of plasma samples or colonic media samples spiked with the studied drugs. Finally, the method was proved to have a high degree of greenness, blueness, and whiteness.

## Electronic supplementary material

Below is the link to the electronic supplementary material.

Supplementary Material 1

Supplementary Material 2

## Data Availability

All data generated or analysed during this study are included in this published article and its supplementary information files.
