# Supplementary material for: Sustainability-based comparative stability of oxaliplatin plus leucovorin and 5-fluorouracil in infusion bags with application to plasma and colonic media samples
Source: Sci Rep. 2025 May 23;15:17982. doi: 10.1038/s41598-025-02079-8 (PMC12102374; doi:10.1038/s41598-025-02079-8)
Supplement: Supplementary file 1 — Supplementary Material 1 [file 41598_2025_2079_MOESM1_ESM.docx]

.

| Drug | Aim of study | Diluent | Temperature | Bag type | Duration of study | Result | Ref |
| --- | --- | --- | --- | --- | --- | --- | --- |
| 5-FU | Study of the Long-term stability of 5-FU stored in ambulatory pump reservoirs and in PVC bags | 5% dextrose and 0.9% sodium chloride | 4 and 21° (bags)  4 or 33°C  (reservoir) | Polyvinylchloride (PVC) bags  / Ethylene vinyl acetate (EVA) or PVC ambulatory pump reservoirs | 14 days | 5-FU was stable in PVC bags for at least 2 weeks at 4 or 21°C.Undiluted 5FU was stable in EVA and PVC reservoirs for 14 days at 33°C. While precipitation is the main factor limiting the storage period at 4°C, it can happen after 3 days in EVA containers and 5 days in PVC ones. | [25] |
|  | Investigation the effect of sterilization process of PVC bags on the 5-fluorouracil stability | 0.9% sodium chloride | 37°C | b-radiation sterilized portable poly(vinyl chloride) (PVC) infusion bags | 14 days | sterilization process of containers influences the stability of 5-FU, particularly decreasing the pH of the drug solution. | [26] |
|  | Determination of the compatibility and stability of 5-FU in 5% glucose diluents and 0.9% sodium chloride filled into the Braun Easypump^Ò^ | 5%glucose and 0.9% normal saline | storage (4^o^C or 25^o^C) and in-use (31^o^C) conditions | Braun Easypump^Ò^ | 21 days | 5-FU showed compatibility and chemical stability in Braun's elastomeric Easy- pump when diluted with 5% glucose or 0.9% sodium chloride for 21 days at 4°C, 25°C, and 31°C, or undiluted at 25°C and 31°C for the same duration. | [27] |
|  | Investigation of the Long-Term Stability of 5-FU following freezing, thawing by microwave and refrigeration | 0.9% sodium chloride | -20^o^C and  5°C ± 3° | Polyvinylchloride (PVC) bags | 107 | The solutions stored in PVC bags remained stable after freezing at –20°C for 79 days followed by storage at 5°C ± 3°C for up to 28 days. | [28] |
|  | Validation of stability of compounded 5-FU in 5% dextrose and 0.9% sodium chloride in different container | 5% dextrose and 0.9% sodium chloride | 25°C and 60% relative humidity /2-8°C | CADD™ cassettes or Intravia™ bags | 15 days | Over 90% of the original concentrations of 5-FU were preserved by the infusions in CADD^TM^ cassettes after 15 days of storage at room temperature and 10 days of refrigeration .The Intravia^TM^ bags demonstrated stability for all compounded 5-FU concentrations for a period of 15 days. | [29] |
| OXA | Examination the oxaliplatin stability in 5% dextrose infusion bags . | 5% dextrose | 3–7 °C / 20–24 °C | polyolefin infusion bags | 30 days | Oxaliplatin in infusion bags with 5% dextrose injection remained chemically stable for at least 30 days at both temperature conditions independent of light exposure. | [30] |
|  | research the physicochemical stability of oxaliplatin dilutions in injections with 5% dextrose kept in infusion bags made of polyvinyl chloride (PVC), polypropylene, and polyethylene. | 5% dextrose | 4 °C and 20 °C | polyvinyl chloride (PVC),polypropylene bag.  Polyethylene bag. | 14 days | oxaliplatin solutions diluted in 5% dextrose injection  remained stable in PVC and PVC-free infusion bags for at least 14 days at both 4 °C and 20 °C without respect to light exposure. | [31] |
|  | Investigation the stability of lyophilized oxaliplatin in  5% dextrose polyolefin infusion bags | 5% dextrose | (2–8 °C) | polyolefin infusion bags | 60 days | lyophilized oxaliplatin infusion solutions kept in 5% dextrose polyolefin infusion bags were chemically unstable after seven days at 2–8°C without light protection, , the concentrated oxaliplatin solution remained steady (above 90%) for a minimum of 60 days.  . | [32] |
|  | Analysis the stability of oxaliplatin in both chloride-containing and chloride-deficient carrier solutions | 5% dextrose 0.9% NaCl Ringer lactate  Dianeal PD4  Phosphate buffer | 42 ^o^C | glass container | 120 min | Oxaliplatin concentration in 5% dextrose remained constant, but deterioration was evident in carrier solutions containing chloride. Oxaliplatin degraded more quickly with increasing chloride concentrations. even in 0.9% NaCl with the highest chloride content, Concentrations were 89.3% of original concentration after 30 minutes and 79.2% of starting concentration after 120 minutes, The concentration of oxaliplatin in Dianeal1 PD4, a commonly used physiological carrier solution, reached 85.3% and 91.7% at 30 and 120 minutes, respectively. | [20] |
| LV | Study of the stability of reconstituted and diluted solutions of lyophilized powdered calcium folinate‏ | 5% dextrose  and 0.9% normal saline | 4^O^C and 23^O^C | PVC bags and in glass bottles | 4 days | The potency of folinic acid slowly decreases at low concentrations, primarily in PVC bags; at higher concentrations, however, the potency of folinic acid remains excellent in both PVC bags and glass bottles. | [33] |
|  | Assessment the long-term stability of sodium folinate in polyolefin bags containing dextrose 5% | 5% dextrose | 4 ^o^C | polyolefin bags | 30 days | sodium folinate solutions have been shown to be stable, since 90% of the original concentration persisted. | [36] |
|  | Analyzing the effects of freezing, microwave thawing, and long-term storage on the stability of calcium levofolinate. | 5% dextrose | 5±3 °C | polyolefin bags | 30 days | Infusions of calcium levofolinate remained stable for one month following freeze-thaw treatment when kept at 5 ± 3 °C. | [37] |
|  | Examination of the stability of calcium folinate in concentrate and diluted forms | 0.9% Normal saline | 15-25^O^C /2-8^O^C | polyethylene (PE) bags and  glass vials | 34 days | When shielded from light, both forms were stable (with less than 10% degradation) for at least 30 days at room temperature and in the refrigerator. | [34] |
|  | Examination of the stability of Calcium levofolinate diluted in infusion bags made of polyolefin/polyamide containing either 5% glucose or 0.9% NaCl. | 5%glucose and 0.9% normal saline | 2-8^O^C | polyolefin/polyamide bags | 14 days | Drug was stable for 14 days, >95% of their initial concentration retained and no change in color and turbidity showed. | [35] |

Table S1 : summary of mobile phases’ composition used for the reported methods for determination of the studied compounds 5-Fluorouracil (5-FU), Oxaliplatin (OXA), and Leucovorin (LV)

| **Analyzed drug** | **Matrix /aim of study** | **Mobile phase system** | | **Runtime**  **(min)** | **Ref** |
| --- | --- | --- | --- | --- | --- |
|  |  | **Type of elution** | **Mobile composition** |  |  |
| ternary mixture of 5-FU, OXA and LV | Study the stability of a ternary mixture of 5-FU, OXA and LV at 3 different pHs values and two concentrations. | Gradient elution | methanol and deionized water (pH , 4.5) was adjusted with trifluoroacetic acid (0.001% v/v) | 68 | [17] |
| 5-FU /LV | Simultaneous Quantification of 5-FU and LV in Human Spiked Plasma and Pharmaceutical Dosage Form | Isocratic elution | 20 mM KH_2_ PO_4_ buffer and methanol (80:20,V/V) with pH 5.4 . | 15 | [39] |
| 5-FU /OXA | Simultaneous determination of 5-FU and OXA in new biodegradable nanoparticles | Isocratic elution | 0.02 M phosphate buffer ,pH 4 and methanol (90:10,V/V) | 12 | [40] |
| 5-FU | Study the Long-Term Stability of 5-FU in 0.9% normal saline) after freezing, thawing by microwave and refrigeration | Isocratic elution | 5% methanol and 95% aqueous 0.01 M potassium dihydrogen phosphate was adjusted to pH 7.50 with aqueous 5.0 M NaOH | 3 | [28] |
|  | Determination of the stability of 5-FU in Intravia™ bags and CADD™ cassettes stored at refrigeration (2-8°C) and room temperature | Isocratic elution | 5 mM potassium phosphate buffer ,pH 6 and methanol(70:30,v/v) | 4 | [29] |
|  | Examination of the stability of 5-FU in rat caecal content as a simulated colon medium. | Isocratic elution | 40 mM phosphate buffer adjusted to pH 7.0 with 10% w/v KOH | 10 | [41] |
| OXA | Investigation the stability of OXA in 5% dextrose infusion bags | Isocratic elution | 0.6 mM phosphate buffer adjusted to pH 3.0 with aqueous 1 N HCl and acetonitrile (99:1 ,v/v) | 15 | [31] |
|  | Comparison stability of OXA in chloride-containing carrier solutions with chloride-deficient carrier solutions | Isocratic elution | 10% of acetonitrile in water for injection ,pH 3.0 using ortho-phosphoric acid | 12 | [20] |
|  | Study of the in-use stability of lyophilized Oxaliplatin and lactose formulation diluted in infusion bag of 5% dextrose | Isocratic elution | 0.002 M monobasic sodium phosphate and phosphate acid in HPLC grade water , pH 3.5 was adjusted with 1N HCl and mixed with acetonitrile (99 : 1, v/v). | 10 | [32] |
|  | Examination of the stability of extemporaneous dilutions of oxaliplatin in polyvinyl chloride, polypropylene, and polyethylene infusion bags containing 5 % dextrose | Isocratic elution | 10% methanol /water (90:10,v/v) and 5 mM hexane sulfonate buffer (pH 3.4). | 15 | [30] |
| LV | Assessment the stability of LV in concentrate in glass vials and diluted in normal saline in polyethylene bags at 2-8^O^C and 15-25^O^C | Isocratic elution | 20 mM Ammonium acetate, pH 3.4 (adjusted with acetic acid) and acetonitrile (95 : 5, v/v) | 10 | [34] |
|  | Study of the stability of LV diluted in 0.9% NaCl and 5%glucose in polyolefin/ polyamide infusion bags and reconstituted in syringes | isocratic elution | acetonitrile and water (60:40, v/v) adjusted to pH 4.8 with 20 mM ammonium acetate buffer | 7 | [35] |
|  | Examination of the effects of long-term storage at 5±3 °C, freezing, and microwave on the stability of LV in a 5% dextrose solution. | isocratic elution | methanol and KH2PO4 (95 : 5, v/v) adjusted to appropriate pH with 5 M NaOH | 5 | [37] |

Table S2: Analytical parameter for the determination of 5-Fluorouracil (5-FU), Oxaliplatin (OXA), and Leucovorin (LV) ternary mixtures using the proposed HPLC method.

| Ratio  (5FU/OXA/LV) | Nominal value, µg/mL | | | Recovery, % ± SD | | | E*_r_* % | | |
| --- | --- | --- | --- | --- | --- | --- | --- | --- | --- |
|  | 5-FU | OXA | LV | 5-FU | OXA | LV | 5-FU | OXA | LV |
| 1:2:1 | 5 | 10 | 5 | 100.93  ±0.08 | 99.76  ±0.06 | 100.8  ±0.08 | 0.93 | -0.02 | 0.8 |
| 1:1:3 | 10 | 10 | 30 | 99.63  ±0.06 | 99.83  ±0.1 | 100.16  ±0.21 | -0.37 | -0.16 | 0.16 |
| 3:1:1 | 24 | 8 | 8 | 100.06  ±0.06 | 99.83  ±0.03 | 99.93  ±0.01 | 0.06 | -0.16 | -0.07 |
| 50:3:14 | 50 | 3 | 14 | 100.26  ±0.27 | 99.33  ±0.02 | 99.96  ±0.01 | 0.26 | -0.67 | -0.04 |

Table S3: Determination of 5-fluorouracil (5-FU), Oxaliplatin (OXA), and Leucovorin (LV) in laboratory-prepared mixtures using the proposed HPLC method.( E*_r_* %: Percentage relative error, SD: standard deviation )

| Interday | | Intraday | | Theoretical  concentration  (µg/mL) | Compound |
| --- | --- | --- | --- | --- | --- |
| RSD, % | Mean  Concentration  (µg/mL) | RSD, % | Mean  Concentration  (µg/mL) |  |  |
| 0.5 | 40.24 | \| 0.17 \| \| --- \| | \| 39.93 \| \| --- \| | 40 | 5-FU |
| 0.3 | 25.13 | \| 0.2 \| \| --- \| | 25.11 | 25 |  |
| 0.04 | \| 0.49 \| \| --- \| | 0.04 | 0.49 | 0.5 |  |
| 0.8 | 100.79 | \| 0.5 \| \| --- \| | 99.89 | 100 | OXA |
| 0.2 | 20.52 | 0.11 | 20.25 | 20 |  |
| \| 0.09 \| \| --- \| | \| 1.01 \| \| --- \| | 0.08 | 1.01 | 1 |  |
| 0.9 | 90.38 | 0.7 | 89.97 | 90 | LV |
| 0.25 | 20.91 | 0.2 | 19.97 | 20 |  |
| 0.08 | 0.93 | 0.06 | 1.11 | 1 |  |

Table S4: Summary of intraday (repeatability) and interday (intermediate precision) precision data for simultaneous determination of 5-Fluorouracil (5-FU), Oxaliplatin (OXA), and Leucovorin (LV) by the proposed HPLC method( RSD: relative standard deviation) .

| Parameter | Mean %recovery ±SD | | | RSD % | | | *t*_r (min)_ ±SD | | |
| --- | --- | --- | --- | --- | --- | --- | --- | --- | --- |
|  | 5-FU | OXA | LV | 5-FU | OXA | LV | 5-FU | OXA | LV |
| Detection Wavelength (±2 nm)  264,266,268  252,254,256  286,288,290 | 99.83±0.11 |  |  | 0.11 |  |  | 3.30 ± 0.03 |  |  |
|  |  | 100.59±0.08 |  |  | 0.77 |  |  | 4.04 ±0.003 | \|  \| \| --- \| |
|  |  |  | 100.00 ±0.4 |  |  | 0.4 |  |  | 10.2 ±0.4 |
| p H of acidified water (±0.2)  4.1,4.3,4.5 | 99.85 ±0.09 | 100.25±0.29 | 99.7±0.17 | 0.09 | 0.29 | 0.17 | 3.21 ± 0.19 | 4.02 ±0.03 | 10.3 ± 0.3 |

Table S5: Evaluation of the robustness of the proposed HPLC method for the determination of 5-Fluorouracil (5-FU), Oxaliplatin (OXA), and Leucovorin (LV). (Mean %recovery of the mixture containing 50 µg/mL 5-FU, 3 µg/mL OXA, and 14 µg/mL LV).

|  | t*_R_* _(min)_ | N | α | R_S_ | T |
| --- | --- | --- | --- | --- | --- |
| 5-FU | 3.324 | 9604 |  | | 1.16 |
|  |  |  | 1.22 | 5.5 |  |
| OXA | 4.06 | 14400 |  | | 1.25 |
|  |  |  | 2.65 | 24.75 |  |
| LV | 10.084 | 11236 |  | | 0.94 |

Table S6: Performance characteristics of the proposed HPLC method for the determination of 5-Fluorouracil (5-FU), Oxaliplatin (OXA), and Leucovorin (LV). (Reference values Theoretical plates (N) >2000 ,Selectivity factor (α) >1 ,Resolution (Rs) >1.5 and Tailing factor (T) ≤ 2)

| Binary mixture* (Oxaliplatin) in NS (RT) | | | | | | | | | | | | | | | |
| --- | --- | --- | --- | --- | --- | --- | --- | --- | --- | --- | --- | --- | --- | --- | --- |
| Time (hour) | 0 | 0.5 | 1 | 1.5 | 2 | 2.5 | 3 | 3.5 | 4 | 4.5 | 5 | 5.5 | 6 | 6.5 | 7 |
| %intact drug | 100.00 | 98.98 | 97.97 | 96.45 | 93.91 | 92.39 | 90.36 | 86.80 | 81.22 | 78.17 | 77.16 | 73.10 | 72.08 | 71.07 | 70.56 |
| conc (µg/mL) | 3.00 | 2.96 | 2.93 | 2.89 | 2.81 | 2.77 | 2.71 | 2.60 | 2.43 | 2.34 | 2.31 | 2.19 | 2.16 | 2.13 | 2.11 |
| Binary mixture* (Oxaliplatin) in NS (2-8^O^ C) | | | | | | | | | | | | | | | |
| Time (hour) | 0 | 0.5 | 1 | 1.5 | 2 | 2.5 | 3 | 3.5 | 4 | 4.5 | 5 | 5.5 | 6 | 6.5 | 7 |
| %intact drug | 100.00 | 99.72 | 99.15 | 98.86 | 97.33 | 95.45 | 94.49 | 93.18 | 92.67 | 92.05 | 91.82 | 91.08 | 90.91 | 90.6 | 90.34 |
| Conc (µg/mL) | 3.00 | 2.99 | 2.97 | 2.96 | 2.91 | 2.86 | 2.83 | 2.79 | 2.78 | 2.76 | 2.75 | 2.73 | 2.72 | 2.72 | 2.71 |
| Single^**^ (Oxaliplatin) in NS (RT) | | | | | | | | | | | | | | | |
| Time (hour) | 0 | 0.5 | 1 | 1.5 | 2 | 2.5 | 3 | 3.5 | 4 | 4.5 | 5 | 5.5 | 6 | 6.5 | 7 |
| %intact drug | 100.00 | 99.23 | 98.16 | 97.96 | 94.39 | 93.37 | 90.81 | 88.26 | 85.20 | 82.65 | 81.12 | 79.08 | 77.04 | 76.02 | 75.51 |
| Conc (µg/mL) | 3.00 | 2.97 | 2.94 | 2.93 | 2.83 | 2.80 | 2.72 | 2.65 | 2.55 | 2.47 | 2.43 | 2.37 | 2.31 | 2.28 | 2.26 |
| Single ^**^(Oxaliplatin) in NS (2-8^O^ C) | | | | | | | | | | | | | | | |
| Time (hour) | 0 | 0.5 | 1 | 1.5 | 2 | 2.5 | 3 | 3.5 | 4 | 4.5 | 5 | 5.5 | 6 | 6.5 | 7 |
| %intact drug | 100.00 | 99.73 | 99.45 | 99.07 | 98.41 | 98.08 | 97.53 | 94.79 | 94.25 | 93.92 | 93.70 | 93.59 | 93.32 | 93.15 | 92.60 |
| Conc (µg/mL) | 3.00 | 2.99 | 2.98 | 2.97 | 2.95 | 2.94 | 2.92 | 2.84 | 2.82 | 2.817 | 2.81 | 2.80 | 2.799 | 2.794 | 2.77 |
| Binary mixture*(Oxaliplatin) in D5W (RT) | | | | | | | | | | | | | | | |
| Time (hour) | 0 | 0.5 | 1 | 1.5 | 2 | 2.5 | 3 | 3.5 | 4 | 4.5 | 5 | 5.5 | 6 | 6.5 | 7 |
| %intact drug | 100.00 | 100.00 | 100.00 | 98.21 | 92.68 | 89.29 | 88.11 | 83.93 | 78.21 | 77.68 | 77.12 | 74.64 | 72.50 | 71.96 | 71.70 |
| Conc (µg/mL) | 3.00 | 3.00 | 3.00 | 2.94 | 2.78 | 2.67 | 2.64 | 2.51 | 2.34 | 2.33 | 2.31 | 2.23 | 2.17 | 2.158 | 2.15 |
| Binary mixture* (Oxaliplatin) in D5W (2-8^O^ C) | | | | | | | | | | | | | | | |
| Time (hour) | 0 | 0.5 | 1 | 1.5 | 2 | 2.5 | 3 | 3.5 | 4 | 4.5 | 5 | 5.5 | 6 | 6.5 | 7 |
| %intact drug | 100.00 | 99.85 | 99.71 | 99.07 | 98.93 | 98.06 | 97.52 | 97.09 | 96.75 | 95.39 | 94.42 | 93.98 | 93.69 | 92.96 | 92.23 |
| Conc (µg/mL) | 3.00 | 2.995 | 2.991 | 2.97 | 2.96 | 2.94 | 2.92 | 2.91 | 2.90 | 2.86 | 2.83 | 2.82 | 2.81 | 2.78 | 2.76 |
| Binary mixture* (Leucovorin) in D5W (RT) | | | | | | | | | | | | | | | |
| Time (hour) | 0 | 0.5 | 1 | 1.5 | 2 | 2.5 | 3 | 3.5 | 4 | 4.5 | 5 | 5.5 | 6 | 6.5 | 7 |
| %intact drug | 100.00 | 100.00 | 100.00 | 100.00 | 100.00 | 100.00 | 100.00 | 100.00 | 100.00 | 100.00 | 98.35 | 98.35 | 98.35 | 98.35 | 98.22 |
| Conc (µg/mL) | 14.40 | 14.40 | 14.40 | 14.40 | 14.40 | 14.40 | 14.40 | 14.40 | 14.40 | 14.40 | 14.16 | 14.16 | 14.16 | 14.16 | 14.14 |

Table S7: Detailed stability data for the selected compounds in normal saline (NS) or Dextrose (D5W) over a period of 7 hours. ^*^Stability was performed on a binary mixture of Oxaliplatin (OXA), and Leucovorin (LV) in infusion bag, equivalent to measured solution (OXA) 3 µg/mL, and (LV) 14.4 µg/mL.

^**^ Stability was performed on single Oxaliplatin (OXA) in infusion bag, equivalent to measured solution of OXA ( 3 µg/mL).

| Parameters | 5-FU | Oxa | LV |
| --- | --- | --- | --- |
| Detection wavelength (nm) | 266 | 254 | 288 |
| Linearity range (µg/ml) | 0.6-20 | 0.5-30 | 0.2-20 |
| Intercept (a) | 7.7 | 0.25 | -7.59 |
| Slope (b) | 74.2 | 7.39 | 1174.98 |
| Correlation coefficient (r) | 0.999 | 0.999 | 0.999 |
| R ^2^ | 0.999 | 0.999 | 0.999 |
| s_a_ | 6.5 | 0.33 | 14.87 |
| s_b_ | 1.14 | 0.11 | 11.88 |
| S_y/x_ | 10.9 | 0.54 | 20.27 |
| F | 4179.20 | 4023.03 | 9768.06 |
| Significance F | 3.43 X 10^-7^ | 3.7X 10^-7^ | 2.28 X 10^-6^ |

Table S8: Analytical parameters for the determination of 5-Fluorouracil (5-FU), Oxaliplatin (OXA), and Leucovorin (LV) ternary mixtures spiked in plasma using the proposed HPLC method (S_y/x_, standard deviation of residuals; S_a_, standard deviation of intercept; S_b_, standard deviation of slope; F, variance ratio, equals the mean of squares due to regression divided by the mean of squares about regression (due to residuals); LOD, limit of detection; LOQ, limit of quantitation).

| Parameters | 5-FU | Oxa | LV |
| --- | --- | --- | --- |
| Detection wavelength (nm) | 266 | 254 | 288 |
| Linearity range (µg/ml) | 0.6-20 | 0.5-30 | 0.2-20 |
| Intercept (a) | 5.33 | 1.21 | 18.87 |
| Slope (b) | 78.09 | 7.56 | 777.14 |
| Correlation coefficient (r) | 0.999 | 0.999 | 0.999 |
| R ^2^ | 0.998 | 0.999 | 0.999 |
| s_a_ | 9.06 | 0.24 | 11.50 |
| s_b_ | 1.58 | 0.11 | 8.16 |
| S_y/x_ | 15.06 | 0.33 | 19.12 |
| F | 2423.709 | 4308.63 | 9064.15 |
| Significance F | 1.02 x 10^-6^ | 7.79 x 10^-6^ | 7.3x 10^-8^ |

Table S9: Analytical parameters for the determination of 5-Fluorouracil (5-FU), Oxaliplatin (OXA), and Leucovorin (LV) ternary mixtures spiked in colonic media using the proposed HPLC method (S_y/x_, standard deviation of residuals; S_a_, standard deviation of intercept; S_b_, standard deviation of slope; F, variance ratio, equals the mean of squares due to regression divided by the mean of squares about regression (due to residuals); LOD, limit of detection; LOQ, limit of quantitation).
