# Supplementary material for: Sustainability-based comparative stability of oxaliplatin plus leucovorin and 5-fluorouracil in infusion bags with application to plasma and colonic media samples
Source: Sci Rep. 2025 May 23;15:17982. doi: 10.1038/s41598-025-02079-8 (PMC12102374; doi:10.1038/s41598-025-02079-8)
Supplement: Supplementary file 2 — Supplementary Material 2 [file 41598_2025_2079_MOESM2_ESM.docx]

5 FU

a)

OXA

b)

LV

c)

a ‘)

a’’)

b ‘)

b’’)

c’’)

Fig. S1 Typical chromatograms of a studied mixture of 5-Fluorouracil (5-FU) (5 µg/mL),OXA (25 µg/mL),and LV (8 µg/mL) using optimized chromatographic conditions measured at 266 nm (a), 254 nm (b), and 288 nm (c), for the determination of 5-FU ,OXA, and LV ,respectively, their corresponding absorption spectra (a'-c') and their peak purity graphs (a"-c") .

| 1. Dextrose Day zero RT   Single 5-FU | a’) Dextrose Day zero (2-8^O^C) |
| --- | --- |
|  OXA  Single OXA |  OXA  Single OXA  5-FU  Single 5-FU |
|  LV  Single LV |  LV  Single LV |
|  |  |
|  OXA  Binary (OXA + LV) |  LV  OXA  Binary (OXA + LV) |

|  | | |
| --- | --- | --- |
| 1. Dextrose Day 4 RT   5-FU  Single 5-FU |  | b^’^) Dextrose Day 4 (2-8^O^C)  5-FU  Single 5-FU |
|  Single OXA |  |  |
| OXA |  | Single OXA  OXA |
|  LV  Single LV |  |  LV  Single LV |
| Binary (OXA+ LV)  |  |  LV  OXA  Binary (OXA+ LV) |

|  | | |
| --- | --- | --- |
| 1. Dextrose Day 7 RT   5-FU  Single 5-FU |  | c^’^) Dextrose Day 7 (2-8^O^C)  5-FU  Single 5-FU |
|  |  |  |
|  OXA  Single OXA  LV  |  |  OXA  Single OXA |
| Single LV  Binary (OXA+ LV)  OXA  LV  OXA |  | LV  Single LV  LV  Binary (OXA+ LV) |

| d) Dextrose Day 9 RT  5-FU  Single OXA  OXA  Single 5-FU |  | d’) Dextrose Day 9 (2-8^O^C)  Single 5-FU  5-FU |
| --- | --- | --- |
|  |  |  |
| Single LV |  | Single LV  Single OXA  OXA |
|  OXAOXA  LV  LV  Binary (OXA+ LV)  LV |  |  OXA  LV  Binary (OXA+ LV)  LV |

OXA

LV

Binary (OXA+ LV)

| 1. Dextrose Day 11 (RT) |  | e’) Dextrose Day 11 (2-8^O^C)  5-FU  Single 5-FU |
| --- | --- | --- |
|  5-FU  Single 5-FU |  |  |
| Single OXA  OXA |  |   OXA  Single Oxa |
|   Single LV  LV |  |   Single LV  LV |
|   Binary (OXA + LV)  OXA  LV |  |  LV  OXA  Binary (OXA + LV) |

| 1. Dextrose Day 14 RT |  | e’) Dextrose Day 14 (2-8^O^C) |
| --- | --- | --- |
| 5-FU  Single 5-FU |  | 5-FU  Single 5-FU |
| LV  Single LV  OXA  Single OXA |  |  OXA  LV  Single LV  Single OXA  Ox |
|  |  | Binary (OXA + LV)  (o  LV  OXA |

LV

Binary (OXA + LV)

(o

OXA

Fig. S2 Typical chromatograms of samples of the studied compounds dispensed in dextrose solution separately equivalent to 5- Fluorouracil (5-FU) (50 µg/mL) , Oxaliplatin (OXA) (3 µg/mL), and Leucovorin (LV) (14.4 µg/mL) /binary mixture of (OXA) (3 µg/mL) and ( LV) (14.4 µg/mL) at (a) day 0, (b) day 4, (c) day 7, (b) day 9 , (e) day 11,(f) day 14 kept at room temperature(RT) and the corresponding chromatograms of refrigerated solutions at 2-8 c a'-f’) measured at 266 nm (5-FU ),254 nm (OXA) and binary mixture (OXA/LV),and 288 nm (LV).

| 1. Normal Saline Day zero RT   Single 5-FU  5-FU | a’) Normal Saline Day zero (2-8^O^C)  Single 5-FU  5-FU |
| --- | --- |
|  |  |
|  OXA  Single OXA |  OXA  Single OXA |
|  Single LV  LV |  Single LV  LV |
|  LVV  OXA  Binary (OXA + LV) |  LV  OXA  Binary (OXA + LV) |

| 1. Normal Saline Day 5 RT   Single 5-FU  5-FU | b’) Normal Saline Day 5 (2-8^O^C)  Single 5-FU  5-FU |
| --- | --- |
|  |  |
|  Single Oxa   OXA | Single Oxa  OXA |
|  Single LV  LV | Single LV  LV |
|  OXA  Binary (Oxa + LV)  LV |  OXA  LV  Binary (OXA + LV) |

| 1. Normal Saline Day 7 RT   Single 5-FU  5-FU | c’) Normal Saline Day 7 (2-8^O^C)  Single 5-FU  5-FU |
| --- | --- |
|  |  |
|    OXA  Single Oxa |  Oxa  Single OXA |
|  Single LV  LV |  LV  Single LV  5-FU |
|  OXA(ND)^**^  LV  Binary (OXA + LV) |  OXA  LVLV  Binary (OXA + LV) |

| d ) Normal Saline Day 12 RT  5-FU  Single 5-FU | d’) Normal Saline Day 12 (2-8^O^C)  5-FU  Single 5-FU |
| --- | --- |
| Single OXA |  OXA  Single OXA |
|  OXA (ND)^**^  Single LV  LV  |  LV  Single LV |
|  OXA(ND)^**^  LV  Binary (OXA + LV) |  OXA  LV  Binary (OXA + LV) |
|  |  |

| 1. Normal Saline Day 14 RT   Single 5-FU  5-FU | e’)Normal Saline Day 14 (2-8^O^C)  5-FU  Single 5-FU |
| --- | --- |
|  |  OXA  Single OXA |
|  OXA (ND)^**^  Single OXA    Single LV  LV | LV  Single LV |
|  | Binary (OXA + LV)  OXA  LV |
| Binary (OXA + LV)  OXA (ND)^**^  LV |  |

Fig.S3 Typical chromatogram of samples of the studied compound dispensed in normal saline solution separately equivalent to 5- Fluorouracil (5-FU) (50 µg/mL) , Oxaliplatin (OXA) (3 µg/mL)and Leucovorin( LV) (14.4 µg/mL) /binary mixture of Oxaliplatin (OXA) (3 µg/mL) and Leucovorin( LV) (14.4 µg/mL) at (a) day 0, (b) day 5, (c) day 7, (b) day 12 , (e) day 14 taken at room temperature(RT) and the corresponding chromatograms of study of stability of refrigerated solutions at 2-8 c a'-e') measured at 266nm for(5-FU ),254nm for(OXA)and binary mixture (OXA/LV),and 288nm for(LV).

^*^(NQ: not quantified but detected)

^**^(ND: not quantified not detected)

| 5-FU  Oxa  IS  LV  a) | |
| --- | --- |
| IS  b) | IS  c) |

Fig.S4 Typical chromatograms of (a), a standard mixture of (5-FU) (3 µg/mL), Oxaliplatin (OXA) (1µg/mL), Leucovorin (LV) (0.74 µg/mL) and internal standard (IS) Trimediazine (80 µg/mL),( b), blank sample spiked with (IS) Trimediazine (80 µg/mL), (c) colonic media, using optimized chromatographic conditions measured at 266 nm.
